# VeGA-RX and VeGA-SCX: Controllable SMARTS-Guided Generative Transformers for Precision-Driven *De Novo* Drug Design

**DOI:** 10.1021/acs.jcim.6c00535

**Published:** 2026-04-17

**Authors:** Pietro Delre, Giada Bellofatto, Antonio Lavecchia

**Affiliations:** Department of Pharmacy, “Drug Discovery Laboratory”, University of Naples Federico II, Via Domenico Montesano 49, I-80131 Naples, Italy

## Abstract

Generative models enable large-scale exploration of chemical space; however, achieving controllable generation that balances novelty with preservation of bioactive features remains challenging. In this study, we introduce a controllable framework based on the VeGA architecture, leveraging SMARTS-RX functional descriptors. We present two distinct SMILES-based generative models: VeGA-RX, conditioned on semantic tokens for flexible exploration, and VeGA-SCX, which integrates topological guidance via Bemis-Murcko scaffolds for high-precision generation. We show that sampling temperature modulates chemical quality, with lower temperatures (*T* = 0.6) reducing structural alerts and improving drug-likeness without additional reinforcement learning (RL). Retrospective benchmarking on five pharmacological targets using a strict leakage-safe holdout strategy reveals a clear functional dichotomy: VeGA-SCX maximizes the recovery of bioactive chemotypes (e.g., >90% on mTORC1), while VeGA-RX effectively navigates complex chemical spaces with superior creativity. Comparative analysis against LDMol, a state-of-the-art text-to-molecule diffusion model, confirms the advantages of our autoregressive approach, which delivers higher chemical validity, stricter adherence to constraints, and significantly lower computational latency. In addition, we validate the framework in data-scarce scenarios targeting DCAF1 and WRN helicase, prioritizing candidate ligands exhibiting stable computational binding modes supported by docking and molecular dynamics simulations. Overall, this framework provides a controllable and interpretable strategy for precision-oriented generative chemistry. The complete workflow is open-source and available on GitHub.

## Graphical abstract

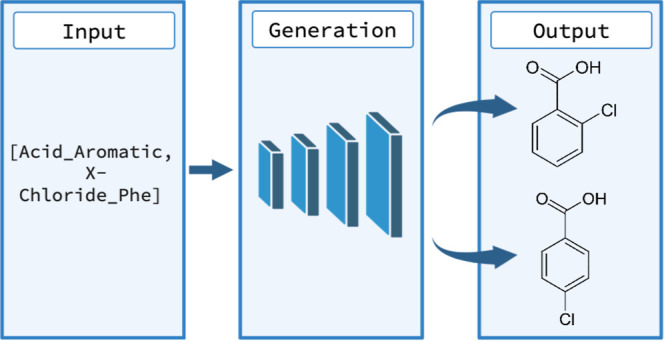

## 1 Introduction

### 1.1 Background

Drug discovery represents a multiobjective optimization problem over an extremely large chemical space. For drug-like small molecules, the number of possible structures is estimated to range from ∼10^23^ to ∼10^60^.^1,2^ Within this vast landscape, identifying compounds that simultaneously meet requirements for potency, target selectivity, and favorable ADME profiles remains resource-intensive and is associated with low success rates throughout the discovery-to-development pipeline.^3^

To move beyond general screening of random compound libraries, the field has progressively adopted rational design and computer-aided strategies. Early computational systems such as LHASA, which was introduced in the early 1970s as a rule-based expert system for synthetic analysis and planning, encoded chemical knowledge as manually defined rules and expert-curated decision logic.^4^

A major shift occurred with the rise of data-driven artificial intelligence approaches, enabling models to learn complex chemical patterns directly from large data sets.^5^ This transition, driven in particular by deep learning, enabled modern generative molecular design, where molecules are generated *de novo*.^6^

Representing molecules as sequences (e.g., SMILES or SELFIES) enabled the adaptation of natural language processing architectures to molecular generation. In this context, the models developed in this work are SMILES-based generative architectures, operating on tokenized molecular sequences within an autoregressive generative framework. While early string-based approaches successfully relied on Variational Autoencoders (VAEs)^7–10^ and Recurrent Neural Networks (RNNs),^11,12^ the field has progressively shifted toward Transformer architectures. By employing self-attention, Transformers more effectively capture long-range dependencies in complex molecular sequences, significantly improving global syntactic coherence.^13^

Consequently, the field’s emphasis has shifted from simply achieving molecular validity to adapting pretrained models for goal-directed design. Current research focuses on conditional and controllable generation, where models are refined through fine-tuning, Reinforcement Learning (RL), or multiobjective optimization to satisfy stringent medicinal chemistry criteria while ensuring chemical validity and synthetic accessibility.^14–16^ In this work, we focus on methodological clarity and rigorous evaluation of controllable generative strategies, avoiding redundant descriptions of standard architectures.

### 1.2 State of the Art

A prominent example of success is REINVENT, a practical workflow in which an RNN-based SMILES generator is first pretrained and then optimized via RL toward user-defined multiparameter objectives.^17^ The later version, REINVENT 4 (R4), supports both RNN and Transformer generators within an RL/transfer-learning framework.^18^

Other decoder-only Transformer architectures developed for *de novo* generation include MolGPT (which explicitly demonstrates conditional generation for property and scaffold control)^19^ and larger-scale GPT-style chemical language models such as ChemGPT, trained with over 1 billion parameters on data sets of up to 10 million unique molecules.^20^

In this landscape, our previously published VeGA model demonstrated that a lightweight, decoder-only Transformer with ∼0.8 million trainable parameters^13^ remains competitive compared to state-of-the-art baselines such as State Space (S4)^21^ sequence models and R4 in target-specific fine-tuning applications.^17,18^ More recently, the field has expanded toward Text-to-Molecule paradigms (e.g., MolT5)^22,23^ and diffusion-based text conditioning, such as TGM-DLM^24^ and the state-of-the-art latent diffusion model LDMol.^25^ Despite this progress, generative text-to-molecule systems face challenges in precision: free-text prompts are often underspecified, which makes it difficult to enforce hard structural constraints. Furthermore, diffusion methods rely on iterative denoising, typically incurring higher inference latency compared to single-pass autoregressive decoding.^22,23,25^ To address these limitations, we introduce a chemically explicit conditioning strategy based on SMARTS-RX descriptors.^26^ By exploiting this hierarchical vocabulary, our framework enables precise, interpretable, and unambiguous constrained generation. To robustly evaluate our approach and its advantages over existing paradigms, we benchmarked it against representative state-of-the-art models from distinct generative frameworks: R4 (RL-optimized generation), S4 (advanced sequence modeling), and LDMol (text-guided diffusion). While standardized benchmarks such as MOSES are widely used for unconditional generation, no universally accepted evaluation framework currently exists for chemically explicit conditional generation tasks.

### 1.3 The VeGA-RX Framework

In this work, we present VeGA-RX and VeGA-SCX, two SMILES-based generative models extending the transformer-based VeGA architecture^13^ designed to combine generative efficiency with precise, chemistry-native conditioning. By utilizing explicit chemical constraints rather than ambiguous free-text prompts, these models support the conditional *de novo* generation of molecules containing precise substructural features, a capability particularly critical in scenarios where fine-tuning is not feasible. To this end, we incorporate SMARTS-RX descriptors (introduced by Kogej et al.), which offer standardized, human-readable definitions of chemical environments.^26^

VeGA-RX utilizes a dual representation during training (SMILES + SMARTS-RX), while VeGA-SCX enriches this paradigm by integrating scaffold-based guidance (SMILES + Scaffold + SMARTS-RX). Our results demonstrate that both models explore chemical space efficiently but with distinct operational profiles: VeGA-SCX prioritizes structural discipline, while VeGA-RX maximizes generative freedom.

We validate this framework through a stepwise analysis, comparing results against the original VeGA baseline. In unconditional generation, we identify sampling temperature as a critical optimization parameter: lowering the temperature (*T* = 0.6) acts as an intrinsic chemical filter, systematically reducing structural alerts and boosting drug-likeness (QED)^27^ providing a computationally lightweight alternative to RL. In comparative transfer-learning experiments across five pharmacological targets: pyruvate kinase muscle isoform 2 (PKM2),^28^ mitogen-activated protein kinase 1 (MAPK1),^29^ glucocerebrosidase (GBA),^30^ mechanistic target of rapamycin (mTORC1),^31^ and farnesoid X receptor (FXR),^32,33^ evaluated under a rigorous leakage-safe retrospective holdout strategy, these architectures demonstrate clear complementarity. VeGA-SCX emerges as a precision tool, maximizing the recovery of bioactive chemotypes (e.g., >90% Recovery Rate on mTORC1), while VeGA-RX excels in exploring a relevant but structurally diverse chemical space. Moreover, benchmarking against LDMol, a state-of-the-art diffusion model, highlights the superiority of our autoregressive framework in terms of strict constraint adherence, chemical validity in complex scenarios, and computational speed.

We demonstrate that these models can be successfully deployed in data-scarce scenarios where fine-tuning is impractical, yet where structural information is accessible through methods such as X-ray crystallography. The efficacy of our approach is driven by the versatility of SMARTS-RX tokens, which enable the generation of diverse analogs satisfying specific pharmacophoric requirements by specifying chemical environments without rigidly prescribing a fixed molecular structure.

Beyond retrospective validation, we demonstrate the prospective applicability of this workflow in data-scarce scenarios, where traditional fine-tuning is unfeasible, using structure-based design supported by molecular docking and molecular dynamics (MD) simulations.

We illustrate this by focusing on two distinct biological systems: DCAF1 (PDB: 8F8E), a substrate receptor for E3 ligases (CRL4 and EDVP) critical for protein degradation and a priority cancer target;^34^ and the Werner syndrome RecQ helicase (WRN, PDB: 8PFO), a synthetic lethal target in microsatellite instability cancers identified by genetic screens, representing a significant unmet medical need despite recent advances in immune checkpoint therapies.^35^ To support reproducibility, the complete source code, pretrained models, and tutorials for both VeGA-RX and VeGA-SCX are freely available in our public GitHub repository (https://github.com/piedelre93/VeGA-RX).

## 2 Methods

### 2.1 Model Development

We implemented a modular end-to-end pipeline for *de novo* molecular generation based on a decoder-only autoregressive Transformer. The pipeline was built in Python 3.9 using RDKit 2022.03 ^36^ and TensorFlow 2.9.0.^37^

### 2.2 Data Set Curation and Preprocessing

#### 2.2.1 Pretraining Data

The pretraining data set was derived from ChEMBL28, which contains ∼2.09 M compounds in its release statistics.^38^ Raw structures were processed through a standardized pipeline consistent with our previous work,^13^ including the handling of salts and mixtures, removal of inorganic structures, removal of duplicates, and stripping of stereochemical information (i.e., resulting in nonisomeric representations). This choice ensures consistency across the ChEMBL data set, where stereochemical annotations are often incomplete or inconsistently specified, and is consistent with common practice in large-scale SMILES-based generative modeling. Under these criteria, the resulting curated set comprised 1,092,285 neutralized and standardized molecules, encompassing 364,673 unique Bemis–Murcko scaffolds. This high scaffold count reflects substantial structural diversity, supporting the coverage of a wide range of chemotypes within the training data.

For each curated molecule, we derived three representations used to construct the training sequences:

1. Target sequence (Y): Nonisomeric canonical SMILES.
2. Scaffold (XScaffold): Bemis–Murcko scaffold, computed with RDKit’s Murcko scaffold utilities.^36^
3. Semantic fingerprint (XSMARTS): Functional-group descriptors derived from SMARTS-RX, a curated hierarchical ontology comprising 406 SMARTS-based functional group definitions.^26^ Across the curated ChEMBL pretraining data set, the average number of SMARTS-RX descriptors per molecule was 4.02 ± 1.44, with a maximum of 12 descriptors observed.

To assess the specific contribution of structural (scaffold) and semantic (SMARTS) constraints, we designed two experimental settings with specific sequence formats:

- SMARTS-only: [SMARTS, < SEP>, Target]
- Hybrid: [SMARTS, < SEP>, Scaffold, < SEP>, Target]

Following VeGA,^13^ we used a custom regex-based tokenizer on these SMILES strings to correctly parse multicharacter atom symbols (e.g., “Cl”, “Br”), ring closures (including multidigit closures), and parentheses. Because Transformer self-attention scales quadratically with sequence length, we analyzed the distribution of composite sequence lengths for the Hybrid configuration

$$L_{tot}=L_{XSMARTS}+L_{XScaffold}+L_{XSMILES}$$

and retained sequences within [5, 140] tokens, chosen to balance computational efficiency and chemical coverage. This maximum length constraint was applied to all experimental settings. After this filtering step, 891,004 SMILES (representing 286,136 unique scaffolds) were retained. Our analysis confirms that this threshold fully captures the distribution of the original ChEMBL28 data set. Specifically, the extremely high ECFP4 centroid similarity (cosine similarity >0.999) between the full and filtered data sets indicates that this step successfully removes outliers while preserving broad chemical space coverage. Together, these results show that the applied filtering preserves both the global chemical space distribution and the underlying scaffold diversity, ensuring that the model is trained on a chemically representative and diverse data set. This constraint primarily removes structurally extreme outliers and does not bias the dominant drug-like chemical space, as confirmed by the high scaffold diversity and global fingerprint similarity reported above. A detailed quantitative comparison of the precurated corpus and the final filtered training set, including selected physicochemical properties and scaffold counts, is reported in the Supporting Information (Table S2).

#### 2.2.2 Fine-Tuning Data Sets

For downstream evaluation, we used the curated low-data benchmarks defined in our repository (https://github.com/piedelre93/VeGA-for-de-novo-design/tree/main/finetuned_models), comprising actives for FXR (882), PKM2 (436), MAPK1 (246), GBA (132), and mTORC1 (77). Each subset was processed using the identical hybrid pipeline to extract SMARTS-RX tokens and scaffolds, ensuring consistent conditioning in transfer learning.

### 2.3 Model Architecture

The model is a decoder-only Transformer for conditional autoregressive generation. It predicts the target SMILES token-by-token conditioned on the concatenated context of SMARTS-RX tokens and (optionally) a scaffold. The architecture comprises 6 masked self-attention layers (6 heads per layer), FFN hidden size 2048, embedding dimension dmodel = 512, and a dropout rate of 0.1. The model utilizes causal masking^39^ and fixed sinusoidal positional encodings.^40^ The full mathematical formulation of the attention mechanism and positional encodings is detailed in the Supporting Information (Section S1).

#### 2.3.1 Training Protocol

We trained using the Adam optimizer with the standard Transformer inverse square-root warmup schedule.^41^ The complete mathematical formulation of this schedule is detailed in the Supporting Information (Section S1). Data were split into 90% training and 10% validation sets. The objective was sparse categorical cross-entropy with an explicit mask excluding <PAD> tokens from gradient updates. Training ran for up to 100 epochs.

#### 2.3.2 Curriculum Learning and SMILES Augmentation

We employed curriculum learning to stabilize training dynamics by ordering molecules according to topological complexity (quantified via ring count, i.e., Smallest Set of Smallest Rings, and branching statistics), progressively introducing more complex structures as validation improvement plateaued.^42^ Additionally, we applied on-the-fly SMILES augmentation: with a probability of 0.1, the target canonical SMILES was replaced by a randomized equivalent. This probability was selected based on our previous systematic analysis, which identified 0.1 as the optimal compromise between exploring chemical diversity and maintaining high distributional fidelity.^13,43^

### 2.4 Conditional Generative Process

The conditioning context C is formally defined as the tuple C ≔ (X_SMARTS, X_Scaffold), where X_SMARTS represents the set of tokens describing the required functional environments and X_Scaffold is the SMILES string of the structural core to be preserved. This formulation supports flexible usage scenarios, ranging from functional-group-guided *de novo* design (where X_Scaffold is empty) to scaffold decoration.

Given a conditioning context *C* and a target SMILES sequence *Y* = (*y*_1, ..., *y*_*m*) of length *m*, the model approximates the joint conditional probability of the sequence autoregressively

1 $$P\left(Y|C\right)=\prod_{t=1}^{m}P\left(y_{t}|y_{<t},C;\theta \right)$$

In this formulation, *y*_*t*_ denotes the token generated at step *t*, *y*_<*t*_ represents the sequence history up to step *t* – 1, and θ corresponds to the learnable parameters of the Transformer. The product operator ∏ indicates that the probability of the complete sequence is factorized autoregressively into the conditional probabilities of each successive token.

#### 2.4.1 Inference and Prompt Construction

At inference time, the model is primed with a prompt sequence *S*_prompt_ constructed to match the specific conditioning mode required by the user, consistent with the training formats described in Section 2.2. The prompt is built starting with a [START] token, followed by the context constraints and their delimiters:

1. SMARTS tokens (if provided) are appended, followed by a [SEP] delimiter.
2. Scaffold tokens (if provided) are tokenized via the regex tokenizer and appended, followed by a second [SEP] delimiter.

For example, in a Hybrid setting, the prompt takes the form

2 $$S_{prompt}=\left[START\right]⊕X_{SMARTS}⊕\left[SEP\right]⊕X_{Scaffold}⊕\left[SEP\right]$$

where ⊕ denotes the sequence concatenation operator. In an unconstrained setting, the prompt simplifies to just [START]. The model then generates the molecule by predicting the subsequent tokens until the [END] token is produced or the maximum length is reached.

#### 2.4.2 Sampling Strategy

Decoding proceeds token-by-token. To balance exploration and exploitation, the next-token distribution is modulated via temperature scaling before sampling

3 $$P\left(w_{i}|w_{<i}\right)=softmax\left(\frac{z_{i}}{T}\right)$$

where *z*_*i*_ represents the logit vector predicted by the model at step *i*, and *T* is the sampling temperature hyperparameter. In our experiments, we typically used *T* = 1.0 for standard generation, though this parameter can be adjusted to control the stochasticity of the output.

### 2.5 Transfer Learning for Target-Specific Generation

To assess the model’s capacity for targeted molecular design and to evaluate target-specific adaptation and rediscovery capability under a leakage-safe split, we implemented a comprehensive fine-tuning protocol. This setup was specifically designed to simulate a low-data regime typical of early stage drug discovery. In this context, both data set size and composition, in terms of scaffold diversity and chemotype distribution, influence the difficulty of the task and the interpretation of performance metrics.

For each of the five biological targets, a leakage-safe holdout set was constructed via a two-step partitioning strategy. First, the data set of active compounds was split based on Bemis–Murcko scaffolds. Second, to ensure structural distinctiveness, we applied sphere exclusion filtering: any molecule in the preliminary test set exhibiting a Tanimoto similarity (ECFP4, 2048 bits) ≥0.6 to any training molecule was removed.

The similarity threshold of 0.6 was not arbitrarily selected but follows established evaluation protocols in prior VeGA and S4 studies, where it was shown to provide a meaningful compromise between near-duplicate recovery (≥0.8) and overly permissive similarity (≤0.5).^13,21^ Using this threshold ensures methodological consistency and direct comparability with previously published baselines.

The remaining compounds constituted the final holdout set, reserved exclusively for evaluation. Subsequently, the base VeGA models (pretrained on ChEMBL) were fine-tuned using only the training portion of the split data. This optimization process ran for a maximum of 100 epochs with a reduced learning rate of 5 × 10^–5^ and a batch size of 32. To prevent overfitting on these smaller, target-specific data sets, we employed early stopping based on validation loss.

### 2.6 Evaluation

Generated molecules were evaluated using RDKit^36^ and in-house scripts to assess generative quality, physicochemical fidelity, and target-specific performance. Standard generative metrics included validity, defined as the fraction of generated SMILES strings that could be parsed into chemically valid molecular graphs; uniqueness, defined as the fraction of unique canonical SMILES within the set of valid outputs; and novelty, defined as the fraction of valid and unique molecules not present in the training data set. These metrics are widely adopted as standardized proxies in molecular generative modeling (e.g., MOSES and GuacaMol benchmarks) to assess distributional learning and internal consistency, although they do not directly imply biological activity.^44,45^ However, it is important to note that such distribution-based and heuristic metrics, while useful for benchmarking, have recognized limitations in predicting true biological activity and should be interpreted cautiously in the context of drug discovery.^46^

#### 2.6.1 Physicochemical and Distributional Metrics

We compared key physicochemical properties, including molecular weight (MW), log *P*, ring count, hydrogen bond donors and acceptors (HBD/A), and rotatable bonds. Drug-likeness was assessed using Lipinski’s Rule of Five (Ro5) and PAINS filters. Additionally, we computed the Quantitative Estimate of Drug-likeness (QED) and the Synthetic Accessibility (SA) score.^27,47,48^ The distributional similarity between generated and reference sets was quantified using the Kolmogorov–Smirnov (KS) statistic, Kullback–Leibler (KL) divergence, and Fréchet ChemNet Distance (FCD).^47,49^ To establish a quantitative reference for the transfer-learning tasks, a Baseline FCD was also computed for each target by comparing its specific training set against size-matched random samples drawn from the full ChEMBL pretraining distribution. To mitigate size-related artifacts and ensure statistical robustness across all distributional comparisons (i.e., generated vs training set, and training set vs ChEMBL baseline), we adopted a repeated subsampling protocol. In all cases, we compared random subsets matched to the size of the target-specific training set, repeating this procedure 20 times. Statistical comparisons between temperature conditions were performed using one-way ANOVA and the nonparametric Kruskal–Wallis test.^50,51^ All analyzes were conducted on the full generated data sets (*n* ≈ 9000–9700 molecules per condition). A significance threshold of *p* < 0.05 was adopted.

Final results are reported as the mean ± standard deviation of the computed metrics across these independent runs.^52^

#### 2.6.2 Target-Focused Metrics

For fine-tuning evaluation, performance was quantified using Training Recovery (RR_T), defined as the proportion of fine-tuning training actives rediscovered by the generated library based on at least one ECFP4 Tanimoto similarity ≥0.6 to a training-set molecule; Scaffold Recovery (RS_T and RS_H), defined as the fraction of training or holdout scaffolds represented within the generated library; Similarity to Nearest Neighbor (SNN), defined as the average maximum similarity between each generated molecule and its closest training-set counterpart; and Internal Diversity (IntDiv), quantifying chemical diversity within the generated set.^21^

### 2.7 Chemical Space Visualization

To qualitatively visualize the coverage of the generated chemical space, we employed the Uniform Manifold Approximation and Projection (UMAP) algorithm for dimensionality reduction.^53^ For each target, molecules from the training set, holdout set, and a sample of 10,000 generated compounds were encoded as Morgan fingerprints (ECFP4, 2048 bits, radius 2). The 2D embedding was learned by fitting the model on the combined training and generated data sets; subsequently, the holdout molecules were projected into this established manifold. This projection strategy ensures that the holdout set is accurately contextualized within the chemical space explored by the generative model. The implementation relied on the umap-learn library, utilizing the Jaccard distance metric with *n*_neighbors set to 30 and min_dist to 0.1.

### 2.8 Molecular Docking Simulation

Generated focused libraries were docked into the crystal structures of DCAF1 (PDB code: 8F8E) and the WRN (PDB code: 8PFO).^34,35^ The protein structures were preprocessed using the Protein Preparation Workflow in the Schrödinger Suite 2025-2 ^54^ to add missing hydrogen atoms, rebuild incomplete residues and rings, and assign appropriate protonation states at physiological pH. Ligands were prepared using LigPrep,^55^ enumerating relevant tautomers and protonation states at pH 7.0 ± 2.0. All possible enantiomers of each ligand were also generated. Docking simulations were performed using the Grid-based Ligand Docking with Energetics (GLIDE) protocol, implemented in Schrödinger Suite 2025-2.^56,57^ Full ligand flexibility was allowed, while the receptor was held rigid. The OPLS_2005 force field^58^ was applied with standard precision docking mode. For each target, a cubic grid was centered on the respective cocrystallized ligand (OICR-8268 for DCAF1 and HRO761 for WRN), with inner box dimensions of 10.00 Å and outer box dimensions of 30.00 Å. The inner box constrains the ligand centroid within the experimentally defined binding site, while the outer box defines the maximum volume accessible to ligand atoms, allowing flexible substituents to explore the pocket boundaries without imposing artificial geometric constraints. This setup ensures accurate localization of the ligand core while preserving conformational flexibility of peripheral groups. Docking protocol validity was assessed by redocking the cognate ligands into their binding sites, yielding root-mean-square deviation (RMSD) values of 0.8 Å for DCAF1 and 1.1 Å for WRN (heavy atoms only), which supports the robustness of the docking protocol. These results confirm that the selected grid parameters accurately capture the binding pocket geometry without artificially enlarging the search space.

### 2.9 MD Simulation

To assess the dynamic stability of the predicted binding modes, the top three docking-ranked complexes for both DCAF1 and WRN (six systems in total) were subjected to MD simulations. System preparation was performed using the System Builder tool in Schrödinger Suite 2025-2. The protein–ligand complexes were solvated in an orthorhombic box of TIP3P water molecules with a minimum buffer distance of 10 Å, and the system charge was neutralized with the addition of Na^+^ and Cl^–^ ions to a physiological concentration of 0.15 M. The OPLS4 force field was applied to all atoms. MD simulations were conducted on GPUs using Desmond.^59^ A cutoff of 9.0 Å was used for nonbonded interactions. Prior to production, all systems were minimized and equilibrated using the default relaxation protocol. Production runs were performed under an isothermal–isobaric (*NPT*) ensemble (*P* = 1.01325 bar, *T* = 300 K) using a Nosé–Hoover thermostat^60^ and a Martyna-Tobias-Klein barostat.^61^ Each complex underwent four independent 100 ns MD simulations with a time step of 2.0 fs, initialized with distinct velocity seeds, storing coordinates at 100 ps intervals, resulting in 1000 frames per trajectory (4000 frames total per complex). Postsimulation analysis was carried out using the Simulation Interaction Diagram (SID) tool to compute the RMSD of the ligand and protein Cα atoms and to characterize the persistence of protein–ligand contacts over time. The resulting trajectories showed consistent binding modes and stable key interaction patterns across quadruplicate independent replicates. The use of OPLS_2005 for docking and OPLS4 for MD follows standard Schrödinger workflow practice and ensures methodological consistency. This protocol is consistent with our previous studies.^62,63^ It should be noted that docking scores and MD-derived stability metrics provide qualitative and relative assessments of binding plausibility and are not intended as quantitative predictors of binding affinity, consistent with well-established limitations of structure-based virtual screening approaches.

## 3 Results

This study builds upon the previously published VeGA architecture, a SMILES-based decoder-only Transformer for *de novo* molecular generation. VeGA demonstrated performance on the MOSES benchmark that was competitive with state-of-the-art sequence-based models such as S4 ^21^ and MolGPT.^19^ Furthermore, VeGA accurately reproduced the physicochemical distributions of the ChEMBL chemical space while maintaining adaptability for downstream fine-tuning.

Crucially, its capacity to prioritize bioactive compounds within relevant chemical space was previously validated through retrospective virtual screening on a target-specific case study (FXR), demonstrating statistically significant enrichment relative to random ChEMBL samples, with docking score distributions comparable to experimentally validated actives under identical virtual screening conditions.^13^ To improve clarity, we focus the presentation on key methodological findings, while detailed statistical analyzes are reported in the Supporting Information. Accordingly, benchmarking is structured to separately assess the unconditional generative backbone and the conditional generation framework.

Retaining the statistically validated autoregressive backbone and identical likelihood formulation, we introduce two advanced variants designed for enhanced conditional control: VeGA-RX, which conditions generation on semantic descriptors (SMARTS-RX), and VeGA-SCX, a hybrid model that augments these semantic constraints with structural Bemis–Murcko scaffolds, enabling scaffold-aware generation (Figure 1). Given the increased complexity of these conditional training tasks, the architecture was scaled up relative to the baseline, resulting in a total parameter count of approximately 40 million. Detailed architectural specifications, including a comparison of parameter counts between VeGA and the enhanced variants, are reported in the Supporting Information (Table S1).

**Figure 1:**
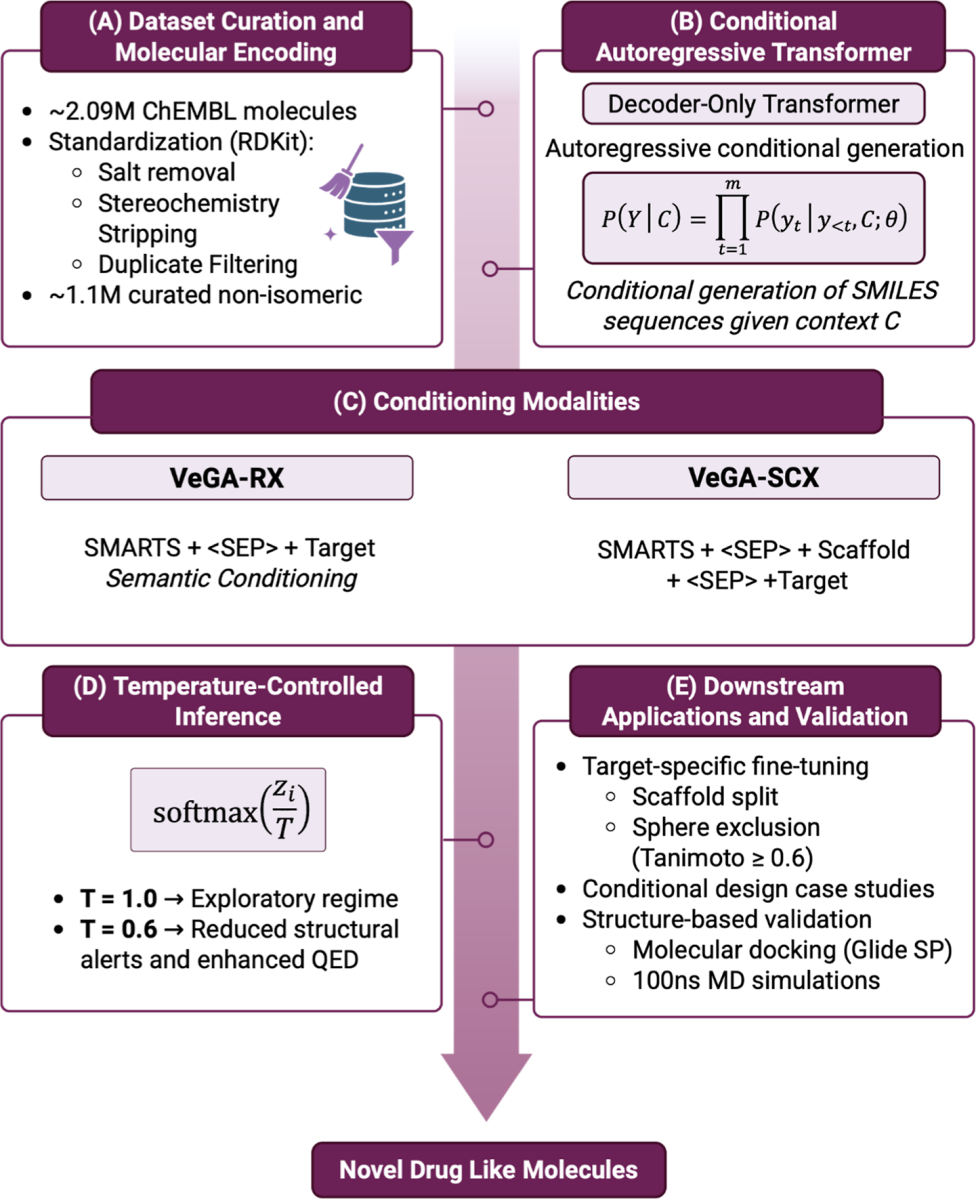
Schematic overview of the VeGA generative framework. The workflow illustrates: (A) data set preprocessing (ChEMBL curation, SMARTS-RX extraction, scaffold computation); (B) the conditional autoregressive Transformer architecture (decoder-only) and the probabilistic modeling of the output sequence; (C) two distinct conditioning modalities: semantic-only (VeGA-RX: SMARTS + SMILES) and hybrid structural-semantic (VeGA-SCX: SMARTS + Scaffold + SMILES); (D) temperature-controlled inference, supporting unconstrained or constrained (SMARTS/scaffold-guided) generation; (E) downstream applications and validation, including target-specific fine-tuning and the final generation of novel drug-like molecules.

In the present study, we systematically benchmark these architectures against VeGA by sampling 10,000 molecules per model. Our evaluation spans five distinct operational domains: first, we assess unconditional generation quality and physicochemical fidelity relative to the ChEMBL chemical space; second, we analyze transfer learning efficiency in low-data fine-tuning scenarios, benchmarking performance against the original VeGA, S4, and R4. We then evaluate the precision of conditional generation driven by structural (Scaffold) and semantic (SMARTS-RX) constraints, followed by a direct comparison with state-of-the-art diffusion-based models (e.g., LDMol) to highlight advantages in validity and efficiency. Moreover, we demonstrate the capability to propose computationally prioritized candidate ligands for data-scarce targets, addressing scenarios where the lack of training examples precludes traditional fine-tuning. For these challenging cases, we implement a rigorous structure-based protocol combining molecular docking, MM-GBSA rescoring, and quadruplicate independent 100 ns MD simulations to confirm the physical plausibility and dynamic stability of the generated candidates.

### 3.1 SMARTS-RX as Descriptors

A critical component of our conditional framework is the adoption of SMARTS-RX as the semantic conditioning vocabulary.^26^ This ontology consists of 406 curated SMARTS patterns specifically designed to capture chemical functions relevant to pharmaceutical reactivity, organized within a three-level hierarchical nomenclature. For our generative strategy, we utilized the Level 3 descriptors (SMARTS-RX), which encode detailed environmental context (e.g., differentiating a chloride on a phenyl ring X-Chloride_Phe from one attached to a heteroaromatic system X-Chloride_Het), ensuring that VeGA is guided by chemically plausible constraints rather than mere substructure presence. To validate the relevance of this vocabulary, we profiled the curated ChEMBL training data set using the SMARTS-RX taxonomy at Level 1 (Class) (Figure S1 A). The distribution reveals the predominance of Nitrogen Heterocycles (75.0%), reflecting their role as privileged scaffolds, followed by linkers like Ethers (43.6%) and Amides (40.3%). Critical functional groups such as Amines (∼25%) and Halogens (21.9%) are also heavily represented, while specific pharmacophoric features include moieties like Sulfonamides (8.0%) and Nitro groups (4.0%). This profile confirms that the training data provides a consistent chemical foundation, ensuring that the model learns to prioritize structures compliant with established medicinal chemistry principles.

### 3.2 Comparative Analysis of Unconditional Generation

We conducted a physicochemical and performance comparison between VeGA and the proposed variants, VeGA-RX and VeGA-SCX, to assess the specific impact of semantic and structural conditioning on the quality, diversity, and drug-likeness of the generated molecules.

While both conditional configurations generate molecules with physicochemical properties broadly consistent with the ChEMBL reference distribution (Figure S2/Table S2 and S3), they exhibit distinct behaviors. VeGA-SCX demonstrates marked structural adherence to the training data, with an average MW closely aligned with the ChEMBL reference (mean 368.90 Da vs 370.77 Da) and a slightly lower average ring count (3.02). In contrast, VeGA-RX shifts toward a slightly higher MW (mean 385.00 Da) and increased structural complexity (mean ring count 3.31 vs 3.22 in the reference). This divergence is a direct consequence of the different conditioning strategies: while VeGA-SCX is structurally guided by a scaffold template, VeGA-RX is conditioned exclusively by semantic descriptions of chemical environments (SMARTS-RX). Consequently, VeGA-RX retains greater generative degrees of freedom to assemble more complex molecular topologies while satisfying functional requirements.

Despite these distinct generative behaviors, both models demonstrate high distributional fidelity. Quantitative analysis based on KS statistics and KL divergence (Table S3) confirms that VeGA-RX and VeGA-SCX closely reproduce the physicochemical distributions of the training set. Notably, in terms of global fidelity (FCD), VeGA-RX achieves a superior score of 0.90 ± 0.02, compared to 0.93 ± 0.02 for VeGA-SCX. Both outperform the original VeGA baseline (0.97 ± 0.02), indicating that the enhanced capacity and conditional training strategy capture the generative chemical space more accurately.

Regarding syntactic validity, performance remains reliable: VeGA-RX achieves 94.40%, while VeGA-SCX maintains 90.10%, with the slight reduction reflecting the increased autoregressive complexity of processing hybrid conditioning sequences. Importantly, this structural discipline does not compromise creativity: both variants maintain uniqueness and novelty scores exceeding 99%, confirming the absence of mode collapse even under strict conditioning.

### 3.3 Impact of Temperature Scaling on Unconditional Generation Quality

We systematically investigated the influence of sampling temperature ranging from 0.6 to 1.0 on the generative process, analyzing a sample size of 10,000 molecules per configuration. Leveraging the established principle that lower temperatures induce a more conservative sampling regime,^64^ we hypothesized that this would favor the selection of high-probability SMARTS-RX descriptors corresponding to drug-like substructures typical of the training distribution, thereby systematically reducing the likelihood of generating structural alerts and undesirable reactive motifs.

This comparison revealed consistent trends: while VeGA exhibited a static performance profile with an average QED constant at 0.56 regardless of sampling temperature, both conditional models demonstrated high sensitivity to temperature tuning. We observed a gradual improvement in drug-likeness and synthetic feasibility for both models. For VeGA-RX, lowering the temperature from 1.0 to 0.6 increased the average QED from 0.56 to 0.65 (Δ ≈ +0.09), while the SA score improved from 2.90 to 2.32. This trend was equally pronounced in VeGA-SCX, where QED rose from 0.60 to 0.68, with a corresponding improvement in SA from 2.86 to 2.38. Furthermore, consistent with a more deterministic sampling strategy, syntactic validity converged to approximately 97% for both models at *T* = 0.6. The negligible temperature sensitivity observed in the baseline VeGA model indicates that the temperature-driven chemical filtering effect emerges from semantic and scaffold conditioning rather than being a generic property of autoregressive sampling.

To elucidate the main reason for this improvement, we performed a comprehensive statistical decomposition of the QED scores, analyzing the weighted contribution of all eight constituent properties to the final metric by comparing data sets generated at *T* = 1.0 and *T* = 0.6 using VeGA-RX as a representative case study (Table S4). A complete breakdown of all QED components, including their statistical significance, is provided in the Supporting Information (Table S4), allowing detailed assessment of the contribution of each descriptor.

While multiple physicochemical properties exhibited statistically significant shifts toward optimal drug-like ranges, this analysis identified the reduction of structural alerts as the primary and most statistically dominant driver (one-way ANOVA *p* < 10^–2^ ^30^): lowering the temperature reduced the mean number of alerts per molecule from 0.93 to 0.46, effectively halving the incidence of reactive motifs.

To map these molecular properties to specific functional groups, we profiled the distribution of SMARTS-RX motifs across data sets generated at *T* = 1.0 and *T* = 0.6 (Figure S1 B,C). Figure 2 and Table S5 highlight the variations most closely associated with structural alerts and QED penalties. Compared to the high-temperature setting (*T* = 1.0), operating at *T* = 0.6 results in a substantial reduction in reactive Michael acceptors (−4.6%), nitro groups (−3.1%), imines (−1.8%), and highly polar moieties such as sulphonamides (−5.4%).

**Figure 2:**
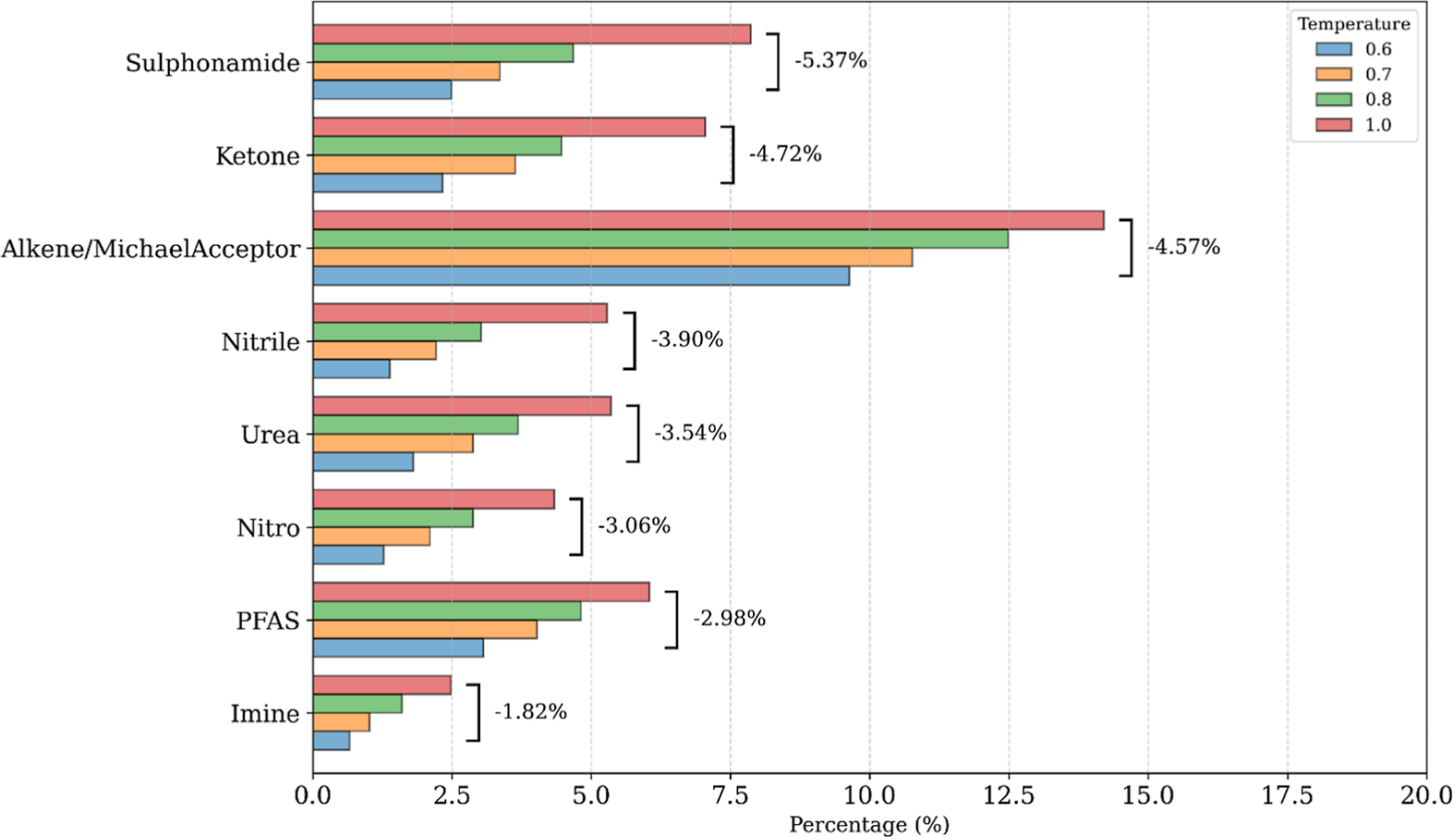
Frequency analysis of selected SMARTS-RX functional descriptors generated by VeGA-RX at sampling temperatures *T* = 1.0 and *T* = 0.6. Bars represent the absolute occurrence frequency of each functional group in the generated data sets. The values reported in square brackets indicate the relative percentage decrease in frequency observed at *T* = 0.6 compared to *T* = 1.0. Lower sampling temperature selectively reduces the incidence of structural-alert-associated motifs, including Michael acceptors, nitro groups, imines, and highly polar functionalities, supporting the role of temperature scaling as an intrinsic chemical filtering mechanism.

This targeted suppression of specific SMARTS-RX categories perfectly correlates with the QED decomposition results, demonstrating that temperature scaling does not merely force the model into a chemically trivial space, but coordinates a targeted redistribution of functional groups. This behavior is consistent with the observed variations across multiple QED components (Table S4), confirming that temperature scaling induces coordinated changes across several physicochemical descriptors rather than affecting a single parameter in isolation.

This trend was confirmed across two additional independent sampling replicates (*n* = 10,000 molecules per replicate), indicating that the effect is not attributable to stochastic variability. Across all runs, the improvement in QED at *T* = 0.6 remained highly significant (one-way ANOVA *p* < 10^–175^; Kruskal–Wallis *p* < 10^–180^), with a similarly significant reduction in structural alerts (*p* < 10^–2^ ^20^). The agreement between parametric and nonparametric tests further supports the robustness of the observed temperature effect. Detailed replicate statistics are reported in Table S6 (Supporting Information).

This finding has important methodological implications for controllable generative design. It demonstrates that, for this specific task, simple temperature scaling acts as an intrinsic “chemical filter”, offering a computationally lightweight alternative to RL frameworks.^15,65^ This optimization does not compromise the originality of the molecules; even at the conservative setting of *T* = 0.6, both models maintain validity, uniqueness, and novelty scores consistently above 99%. Although the larger architecture results in increased generation time compared to the lightweight baseline, rising from ∼20 ms/molecule to ∼220 ms for VeGA-RX and ∼310 ms for VeGA-SCX due to the substantial increase in parameter count (Table S1), this trade-off is strategically advantageous. The ability to directly produce molecular libraries characterized by high QED and SA at lower temperatures positions VeGA-RX and VeGA-SCX as highly efficient engines for generating high-quality candidates ready for downstream computational virtual screening campaigns.

### 3.4 Capturing Bioactivity

To assess the capability for targeted generation, we benchmarked the new conditional architectures against the original VeGA baseline and state-of-the-art models (S4, R4). We adopted the rigorous leakage-safe holdout protocol established in our previous work, generating 10,000 molecules per model for each target. These fine-tuning scenarios represent a rigorous stress test, as the selected targets (mTORC1, MAPK1, PKM2, and GBA) are characterized by a disjointed chemical space where holdout actives are structurally distinct from the training set.^13^ Accordingly, performance should be interpreted not only as a function of data set size but also of chemical-space topology and scaffold composition, which determine whether the task primarily involves interpolation or extrapolation. This setup can be interpreted as a retrospective rediscovery benchmark, where the ability to recover structurally distinct experimentally validated actives provides an indicator of model generalization beyond memorization. In this challenging landscape, the results reveal two distinct operational profiles that share a common advantage: a substantial improvement in retrospective rediscovery metrics compared to VeGA, R4, and S4 (Table 1).

**Table 1:** Performance Comparison of VeGA, VeGA-RX, VeGA-SCX, and Baseline Models (R4, S4) across Five Pharmacological Targets (FXR, GBA, MAPK1, mTORC1, PKM2)^a^

target	data split space profile	model	Val (%)	Uni + Nov (%)	RR_T (%)	RS_T (%)	RS_H (%)	SNN	FCD ±SD	FCD_base_ ± SD
FXR	702/40 continuous	VeGA (base)	94.30	46.64	96.60	57.04	20.80	0.63	3.95 ± 0.15	
		R4	99.10	42.18	96.20	55.63	16.70	0.56	8.34 ± 0.24	
		S4	99.40	23.37	97.39	63.37	25.00	0.75	3.60 ± 0.32	26.12 ± 0.43
		**VeGA-RX**	**93.80**	**68.90**	**89.32**	**46.13**	**25.00**	**0.50**	**5.61** **±** **0.17**	
		**VeGA-SCX**	**88.18**	**29.68**	**96.60**	**66.20**	**16.70**	**0.63**	**3.44** **±** **0.11**	
GBA	104/24 disjointed	VeGA (base)	93.23	82.00	68.18	74.36	10.50	0.37	21.43 ± 0.53	
		R4	99.04	58.28	76.52	80.77	10.50	0.36	25.70 ± 1.11	
		S4	98.00	30.48	78.03	89.74	0.00	0.52	20.24 ± 0.76	36.26 ± 0.98
		**VeGA-RX**	**93.80**	**71.15**	**86.54**	**74.36**	**10.50**	**0.38**	**19.74** **±** **1.14**	
		**VeGA-SCX**	**90.00**	**15.04**	**96.15**	**84.62**	**10.50**	**0.52**	**15.54** **±** **0.92**	
MAPK1	197/49 disjointed	VeGA (base)	94.30	88.83	62.60	48.91	7.00	0.38	13.71 ± 0.32	
		R4	99.28	54.33	69.92	59.24	4.70	0.40	15.09 ± 0.53	
		S4	98.20	65.94	73.92	66.30	9.30	0.46	13.70 ± 0.40	22.28 ± 0.58
		**VeGA-RX**	**93.30**	**68.36**	**80.20**	**59.78**	**7.00**	**0.40**	**12.98** **±** **0.50**	
		**VeGA-SCX**	**87.56**	**38.01**	**88.32**	**82.61**	**4.70**	**0.45**	**11.83** **±** **0.39**	
mTORC1	62/15 disjointed	VeGA (base)	94.30	89.43	75.32	81.63	16.70	0.38	28.07 ± 1.39	
		R4	98.50	72.90	81.82	90.50	16.70	0.32	34.92 ± 1.87	
		S4	98.20	46.66	84.42	89.50	16.70	0.48	29.78 ± 1.52	44.97 ± 1.15
		**VeGA-RX**	**95.80**	**41.01**	**91.94**	**87.76**	**16.70**	**0.43**	**27.01** **±** **2.47**	
		**VeGA-SCX**	**86.07**	**33.66**	**91.94**	**87.76**	**16.70**	**0.47**	**22.55** **±** **1.69**	
PKM2	348/81 disjointed	VeGA (base)	92.40	89.64	46.56	52.48	29.90	0.41	9.46 ± 0.22	
		R4	99.02	55.12	88.07	90.50	29.90	0.46	8.72 ± 0.33	
		S4	98.20	72.19	80.71	78.52	20.90	0.48	9.10 ± 0.31	22.28 ± 0.58
		**VeGA-RX**	**92.80**	**72.57**	**76.44**	**42.13**	**20.90**	**0.42**	**8.93** **±** **0.22**	
		**VeGA-SCX**	**75.22**	**72.28**	**45.69**	**43.26**	**20.90**	**0.36**	**10.10** **±** **0.34**	

a Metrics include validation rate (Val), uniqueness plus novelty (Uni + Nov), RR_T, RS_T and RS_H, SNN, and FCD ±SD. Results highlight complementary operational profiles of RX (exploration) and SCX (precision-driven exploitation). The target-specific FCD_base_ ± SD denotes the intrinsic distance between each training set and ChEMBL, providing a benchmark for distributional matching in small-data regimes.

Specifically, VeGA-SCX acts as a scaffold-inspired generator, maximizing exploitation by utilizing the scaffold as a template. For the challenging mTORC1 target, VeGA-SCX achieves a training RR_T of 91.94%, marking a substantial improvement over the original baseline (75.32%). This trend is even more pronounced for GBA, where it reaches a remarkable RR_T of 96.15%, significantly outperforming the previous state-of-the-art competitor S4 (78.03%). However, this extreme recall entails specific trade-offs: in disjointed spaces, diversity metrics (uniqueness and novelty) drop significantly (e.g., 15.04% for GBA), and for structurally complex targets like PKM2, validity decreases to 75.22%. This behavior reflects the model’s strong bias toward reproducing specific bioactive scaffolds rather than pursuing divergent exploration.

In contrast, VeGA-RX demonstrates surprising versatility: consistent with the greater degrees of freedom afforded by SMARTS-RX, it operates as a flexible explorer while retaining unexpected recovery power. It maintains consistently higher syntactic validity (>92%) and uniqueness and novelty compared to VeGA-SCX. VeGA-RX is not limited to exploration: on mTORC1, it matches the recovery of SCX (91.94%), and on GBA, it achieves a robust 86.54%. Its performance on PKM2 is particularly illustrative: where scaffold constraints hinder SCX (RR_T 45.69%), VeGA-RX leverages semantic guidance to achieve a RR_T of 76.44% (vs 46.56% for VeGA) along with the best distributional fidelity (FCD 8.93). These results suggest that for structurally complex targets, relying solely on semantic features allows the model to bypass the difficulties of replicating intricate scaffolds, successfully generating valid and bioactive analogs where the scaffold-based approach struggles.

Furthermore, beyond RR_T, we evaluated structural generalization by measuring RS_H. Across disjointed targets, RS_H remains consistently nonzero (e.g., 16.7% for mTORC1 and ∼20–30% for PKM2), confirming that generation extends beyond the fine-tuning scaffolds. While VeGA-SCX systematically dominates in training RS_T, reflecting its exploitation capability, VeGA-RX maintains comparable or slightly higher RS_H in several targets, indicating a broader capacity for scaffold hopping toward structurally distinct chemotypes. These observations indicate that generative performance is not determined by data set size alone, but is primarily governed by chemical-space topology and data set composition, particularly in low-data regimes.

Moreover, benchmarking reveals a consistent trend toward improved distributional fidelity across all tested targets following the introduction of conditional constraints. VeGA-SCX generally achieves the lowest FCD scores (e.g., 15.54 for GBA vs 21.43 for VeGA). To appropriately interpret these FCD metrics, they must be contextualized within the target-specific low-data regime. Estimating distributional statistics from highly restricted subsets naturally yields elevated baseline distances due to finite-size statistical effects. Importantly, elevated FCD values in this context should not be interpreted as evidence of mode collapse, but rather as a consequence of limited sample size and increased variance in distribution estimation. As described in the Methods section, we established a quantitative reference by computing a Baseline FCD between each target-specific training set and size-matched random samples from the full ChEMBL distribution. These baseline values quantify the inherent structural divergence of the target-specific actives from the global chemical space (e.g., 44.97 ± 1.15 for mTORC1 and 36.26 ± 0.98 for GBA—Table 1). Consistent with this established reference, we find that the generative models generally achieve FCD values that are substantially lower than their respective baselines; this structural shift demonstrates that the models successfully specialize toward the specific target distributions while maintaining generative diversity.

This behavior is visually corroborated by the UMAP projections (Figure S3): while VeGA-SCX tightly envelops the training set distribution, demonstrating high fidelity to known bioactives, VeGA-RX exhibits a broader projection, covering adjacent regions of unexplored chemical space while maintaining relevance. This confirms that SCX maximizes exploitation, whereas RX successfully bridges the gap between the known training manifold and novel, valid chemical space.

These results highlight distinct strengths across the three architectures: the unconditional VeGA serves broad chemical space sampling; VeGA-RX allows for pharmacophore-biased exploration and scaffold hopping; and VeGA-SCX strictly constrains generation around a predefined core for local optimization. Ultimately, these architectures operate as generators of specialized libraries designed to provide chemically plausible candidates for downstream structure-based evaluation and subsequent in vitro and in vivo experimental validation. In this context, the different VeGA variants should be interpreted as complementary operating modes within a unified generative framework, enabling different levels of constraint depending on the stage of the design process, from exploration to focused optimization.

### 3.5 Conditioned Generation

A key advantage of the proposed framework is the flexibility of its inference modes. VeGA-SCX supports hybrid conditioning, accepting both a structural core (Scaffold) and semantic features (SMARTS-RX), while VeGA-RX operates through pure semantic conditioning. To streamline the workflow, the inference pipeline is designed to accept complex molecular structures as input and automatically extract the Bemis-Murcko scaffold on-the-fly. It is important to note that in VeGA-SCX, the scaffold acts as a soft constraint: rather than performing a rigid copy-paste operation, the model uses the input SMILES as a strong structural template, guiding the generation while retaining the flexibility to introduce slight variations.

To demonstrate these capabilities, we present two representative case studies generated at a sampling temperature of *T* = 1.0. It is worth noting that for these specific illustrative examples, we manually defined simplified scaffolds to isolate and visualize the generative behavior of VeGA-SCX, although the standard pipeline supports fully automated extraction.

First, drawing inspiration from Molecule IA (ChEMBL ID: CHEMBL1397413) (Figure 3), we conditioned VeGA-SCX on a benzene scaffold plus specific SMARTS tokens, while VeGA-RX received only the semantic tokens. Analysis of 1000 generated compounds revealed stable performance: VeGA-SCX achieved 95.1% validity with 98.5% scaffold retention, while VeGA-RX matched this validity with higher uniqueness (94.8% vs 78.8%). Crucially, the explicit benzene constraint in VeGA-SCX successfully focused the generation, limiting the topology to the single benzene ring provided (avg. aromatic rings = 1.0 for SCX vs 2.20 for RX) and resulting in more compact molecules (mean MW 331 Da vs 414 Da for RX).

**Figure 3:**
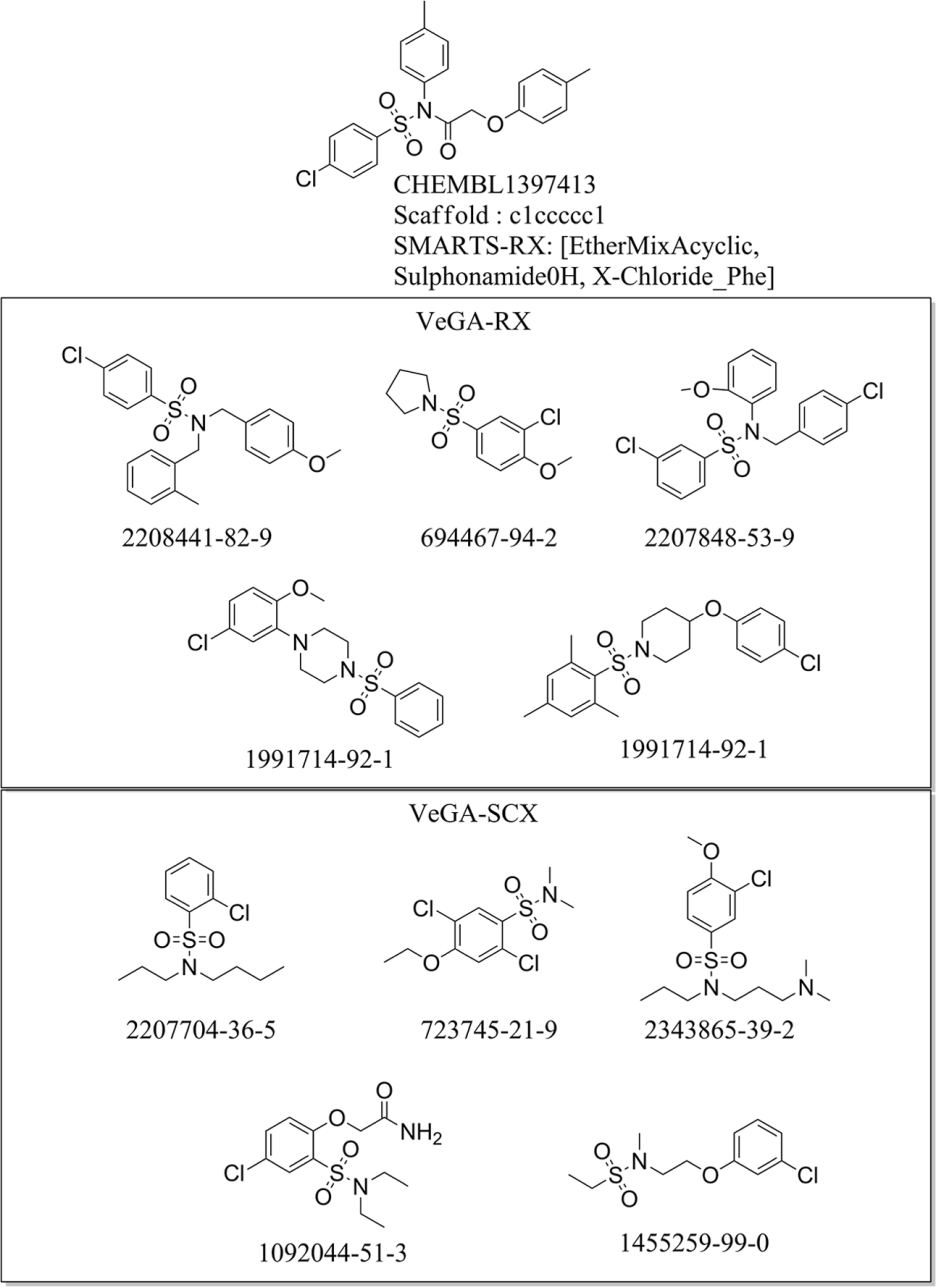
Representative molecules generated under SMARTS-guided and scaffold-guided conditioning inspired by CHEMBL1397413. VeGA-SCX was conditioned on a benzene scaffold combined with SMARTS-RX tokens, whereas VeGA-RX relied exclusively on semantic constraints. The generated compounds highlight the structural discipline enforced by SCX (retention of a single aromatic ring) compared to the expanded topological exploration observed with RX. The numerical identifiers reported below each 2D structure correspond to CAS Finder registry entries, indicating that the generated molecules match existing, synthetically realized compounds.

This dichotomy is further confirmed in the second case study, based on Molecule IA2 (CHEMBL1356585) (Figure S4), which features a benzoxazole core. When conditioned on this scaffold, VeGA-SCX (93.7% validity) maintained the core structure, generating compounds with a 68.9% scaffold match rate and an average aromatic ring count of 2.0. In stark contrast, VeGA-RX, conditioned only on the semantic profile, displayed significantly higher generative creativity. While maintaining high validity (95.9%) and originality (78.1% vs 70.4%), VeGA-RX constructed larger, multiring systems to satisfy the functional requirements (avg. aromatic rings 3.49 vs 2.00 for SCX), leading to a marked increase in MW (ΔMW ≈ +94 Da) and lipophilicity (ΔLogP ≈ + 1.3).

Notably, despite the structural divergence, both models successfully generated molecules that correspond to real, synthesized compounds identified in external databases (yet absent from the ChEMBL training set), providing strong evidence of their chemical relevance and synthetic feasibility. However, VeGA-SCX demonstrated a superior drug-likeness profile in this constrained setting (QED 0.72 vs 0.55 for RX), confirming that providing a structural template effectively focuses generation by exploring specific regions of chemical space. Conversely, the increase in molecular complexity observed with VeGA-RX reflects its unconstrained generative freedom at *T* = 1.0. This behavior is a natural manifestation of the model’s creativity: without a topological constraint, it tends toward structural elaboration to satisfy semantic requirements. However, as demonstrated in our previous analysis, this tendency is not an inherent limitation but a tunable behavior that can be effectively modulated by lowering the sampling temperature.

In terms of computational efficiency, generation remains rapid: creating a batch of 100 molecules requires approximately 30 s for VeGA-SCX and 20 s for VeGA-RX. Ultimately, the choice between the two architectures depends on the strictness of the design requirements: VeGA-SCX offers structural discipline (albeit with probabilistic scaffold retention), while VeGA-RX offers maximum exploratory power, with physicochemical properties that can be fine-tuned via temperature scaling.

### 3.6 Comparison with LDMol, a Diffusion-Based Approach

As a final validation, we contextualized our framework against LDMol, currently regarded as a state-of-the-art diffusion model for conditioned text-to-molecule generation. To minimize ambiguity and ensure a fair comparison, we benchmarked LDMol using the same two case studies described above, providing precise IUPAC names as input prompts. As diffusion-based models are sensitive to sampler configuration, we adopted the default inference parameters reported in the original LDMol study to ensure reproducibility and methodological consistency.^25^ This comparison is intended as a task-level evaluation across representative generative paradigms rather than a standardized benchmark, reflecting the current lack of unified evaluation protocols for conditional molecular generation.

In the first case study (CHEMBL1397413), LDMol achieved an effective validity of ∼71% with a mean QED of 0.43. Property analysis indicates that this reduced drug-likeness is associated with excessive functionalization, including a higher frequency of structural alerts (mean 1.07) and elevated polar surface area (120.1 Å^2^), compared to VeGA-SCX (Alerts 0.40; PSA 51.9 Å^2^) and VeGA-RX (Alerts 0.36; PSA 60.8 Å^2^). Moreover, LDMol generated structures with an average of 3.50 aromatic rings, reflecting a more expansive interpretation of the structural constraint, whereas VeGA-SCX strictly preserved the single-ring topology (1.00 aromatic ring) and VeGA-RX maintained moderate expansion (2.20 rings).

A similar trend was observed in Case 2 (CHEMBL1356585), where LDMol yielded a QED of 0.51 and produced molecules with 1.04 structural alerts on average and a polar surface area of 112.1 Å^2^. In contrast, VeGA-SCX achieved a superior QED of 0.72 with optimized physicochemical properties (PSA 64.7 Å^2^) and strict topological adherence (2.00 aromatic rings vs 3.25 for LDMol). Even VeGA-RX, while exploring larger topologies (3.49 rings), maintained a substantially cleaner structural profile (Alerts 0.12) compared to the diffusion baseline. Notably, while several VeGA-generated candidates matched known compounds in external databases, none of the LDMol outputs were identified in PubChem, suggesting that the explicit constraints in VeGA better navigate the boundaries of known, synthetically relevant chemical space. Furthermore, generating a batch of 100 molecules required approximately 3 min with LDMol, compared to less than 30 s for the VeGA models. These performance gaps suggest that while diffusion models excel in capturing broad semantic distributions, autoregressive models with explicit chemical constraints may offer superior structural coherence for specific scaffold-based tasks.

It is important to note that autoregressive and diffusion-based models rely on fundamentally different conditioning mechanisms. VeGA-RX and VeGA-SCX are not text-native models; they employ explicit chemical token conditioning (SMARTS-RX and scaffold constraints) rather than free-text embeddings. Accordingly, this comparison should be interpreted as a task-level evaluation rather than a strict benchmark. Our objective is not to claim superiority in natural language understanding, but to demonstrate that chemically explicit conditioning provides a reliable and controllable alternative for precision-driven molecular design.

### 3.7 *De Novo* Design for Data-Scarce Targets

To evaluate the practical utility of our models in generating molecules for targets where training data is insufficient for fine-tuning, we focused on two distinct and challenging biological systems: DCAF1 and the WRN helicase. DCAF1, a substrate receptor for CRL4 and EDVP E3 ligases, represents an extreme data-scarce scenario (ChEMBL ID: CHEMBL5465521) with only 28 equilibrium dissociation constant (*K*_d_) data points currently available, making standard transfer learning approaches impractical. Thus, we leveraged the crystal structure of its WDR domain in complex with OICR-8268 (PDB: 8F8E). Similarly, for WRN, emerging discovery strategies prioritize allosteric inhibition, targeting unique interfaces like the D1–D2 junction, to avoid the toxicity risks associated with ATP-competitive cross-reactivity. Consequently, we selected the complex with the allosteric inhibitor HRO761 (PDB: 8PFO) as our second template.

Leveraging these cocrystallized ligands, we extracted their respective Bemis-Murcko scaffolds and SMARTS-RX functional profiles to prompt both VeGA-SCX and VeGA-RX, generating a combined focused library of 10,000 candidates to populate the relevant chemical space surrounding these lead compounds. Following molecular docking, MM-GBSA rescoring was employed to prioritize candidates with the highest theoretical affinity. After expert visual inspection, three top-ranking candidates for each target (Figure 4) were selected for quadruplicate 100 ns MD (4 × 100 ns per complex) simulations to ensure the reproducibility of the binding modes and the stability of key protein–ligand interactions. It is worth noting that the reference ligands possess significant structural complexity (Figure 4A), presenting a nontrivial generative challenge.

**Figure 4:**
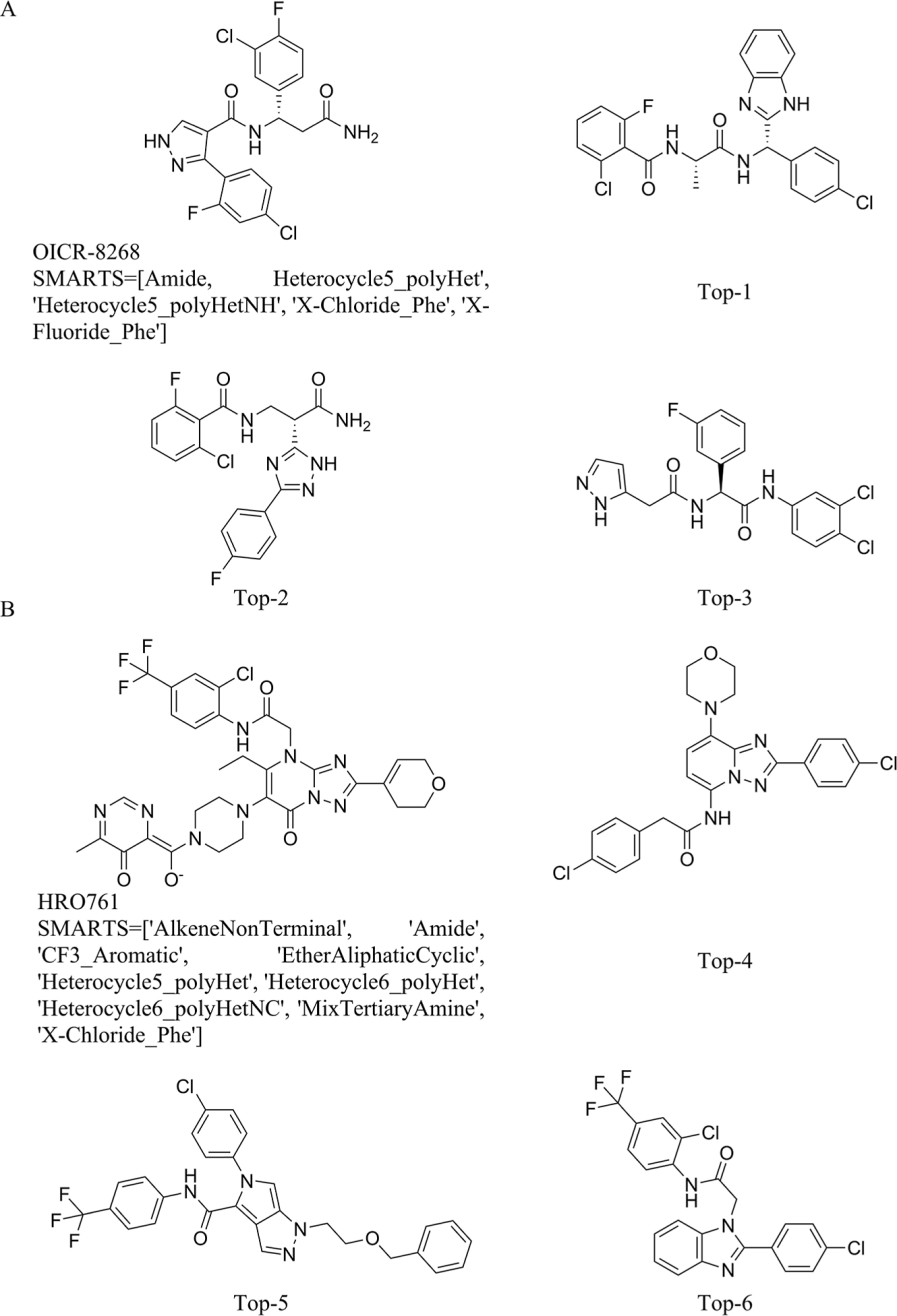
Two-dimensional structures of the reference cocrystallized ligands for A. DCAF1 (PDB: 8F8E) and B. WRN helicase (PDB: 8PFO), together with the top-ranked generated candidates. The Top compounds were selected based on docking score ranking and subsequently subjected to quadruplicate 100 ns MD simulations for binding stability assessment. These structures represent the highest-scoring candidates emerging from the VeGA-RX/SCX-guided focused library generation.

Regarding the DCAF1 system, the reference ligand OICR-8268 exhibited a stable binding mode during the 100 ns simulation (ligand heavy-atom RMSD < 2.0 Å relative to the initial docked pose). Postdocking Prime MM-GBSA rescoring of the docked complexes yielded a Δ*G*_bind of −75.4 kcal/mol for the reference ligand.

The *de novo* compounds showed comparable structural stability, with Top-1 (DS = −9.38 kcal/mol; MM-GBSA = −71.1 kcal/mol), Top-2 (DS = −8.58; MM-GBSA = −63.5 kcal/mol), and Top-3 (DS = −8.12; MM-GBSA = −70.7 kcal/mol) maintaining ligand RMSD values at an average of 3.5 Å across all independent replicates (Figure S5A–C). Notably, Top-1 and Top-3 display MM-GBSA estimates close to that of the reference ligand, supporting a favorable binding pose consistent with the docking model.

To characterize the structural basis of this stability, we evaluated the persistence of interactions maintaining >50% occupancy consistently across the four independent replicates (unless otherwise noted, interactions possess >98% average occupancy). All three candidates successfully conserve the critical interactions defined by the reference ligand (Figure 5/Table S7), stabilizing via a conserved hydrogen bond with Asp1356. Top-1 (Figure 5B) and Top-3 (Figure 5D) exhibit the highest fidelity, maintaining hydrogen bonds with Arg1298 and π–π stacking with Phe1330 (73.1% ± 21.0). Additionally, they extend into a proximal hydrophobic subpocket: Top-1 establishes a stabilizing halogen–hydrogen interaction with the backbone of Phe1101. In contrast, Top-2 prioritizes a strong cation–π interaction with Arg1225 (55.8% ± 4.3).

**Figure 5:**
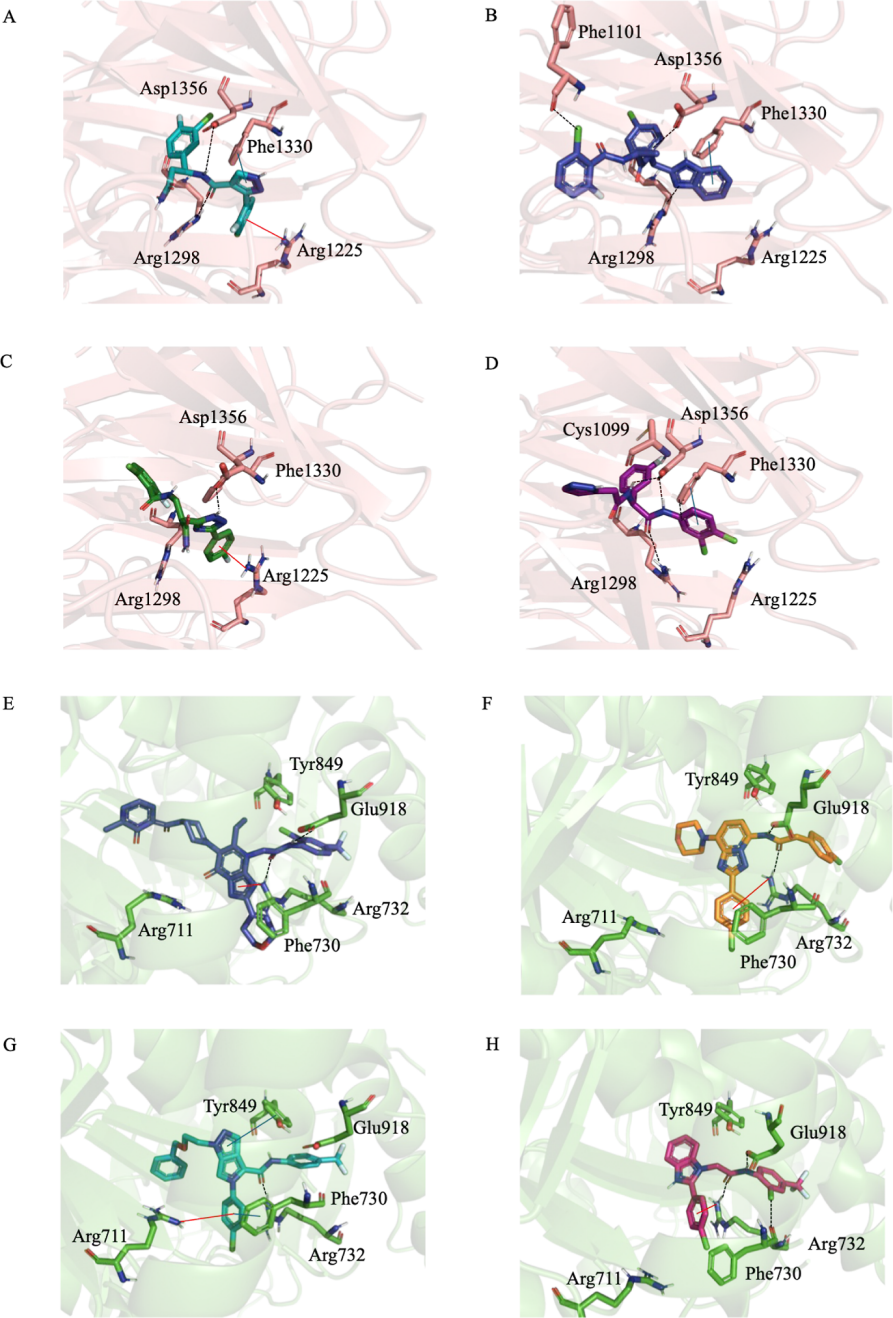
Molecular docking and MD analysis of DCAF1 and WRN helicase complexes. (A–D) DCAF1 system (PDB: 8F8E): Representative frame of the reference ligand OICR-8268 (A) and the three top-ranked candidates, Top-1 (B), Top-2 (C), and Top-3 (D). (E–H) WRN helicase system (PDB: 8PFO): Representative frame of the cocrystallized ligand HRO761 (E) and the three top-ranked candidates, Top-4 (F), Top-5 (G), and Top-6 (H). All representative frames were extracted from the first of the quadruplicate 100 ns MD replicates, showing interactions with an occupancy above 50%. Hydrogen bonds are depicted as black dashed lines, π–π stacking interactions as solid blue lines, and cation–π interactions as solid red lines. Proteins are represented in cartoon format, and ligands are displayed as sticks.

A parallel validation was performed for the WRN system. The reference ligand HRO761 exhibited high structural stability during the 100 ns simulation (ligand heavy-atom RMSD <2.0 Å relative to the initial docked pose). Postdocking MM-GBSA rescoring yielded a Δ*G*_bind of −110.50 kcal/mol.

The generated candidates maintained stable trajectories with ligand RMSD values consistently below 5 Å across the independent runs (Figure S5D–F). Docking and MM-GBSA analysis yielded energetically favorable binding poses for Top-4 (DS = −8.26 kcal/mol; MM-GBSA = −82.29 kcal/mol), Top-5 (DS = −9.97 kcal/mol; MM-GBSA = −77.43 kcal/mol), and Top-6 (DS = −10.28 kcal/mol; MM-GBSA = −75.56 kcal/mol).

Interaction profiling reveals that all three candidates successfully engaged the critical Arg732 residue via stable cation–π and hydrogen bonding interactions (Figure 5—Table S7). Beyond this shared interaction, Top-5 established robust π–π stacking with Tyr849 (55.75% ± 7.85) and Phe730 (67.00% ± 13.74) (Figure 5G); Top-4 mirrored the reference by engaging Glu918; and Top-6 (Figure 5H) successfully replicated the halogen-bonding strategy by establishing a stable interaction between its chlorine atom and the backbone of Phe730 (67.00% ± 13.7), in addition to conserving the Glu918 (99.3% ± 0.3) hydrogen bond.

Collectively, these results indicate that the generative models can propose candidate ligands capable of mimicking the behavior of the cocrystallized ligands while exploring novel binding regions, thereby enabling a deeper investigation of the binding site topologies. These findings should be interpreted as supporting the structural plausibility and prioritization of generated candidates, rather than as direct evidence of binding affinity or biological activity.

## 4 Limitations

Despite the encouraging results, several limitations should be acknowledged. The framework relies on predefined SMARTS-RX descriptors and optional scaffold constraints, meaning that its performance depends on the quality and relevance of user-defined chemical prompts. While this design ensures interpretability and precise controllability, it does not directly encode protein structural information and therefore requires prior knowledge of the target’s chemical or structural features to define appropriate constraints.

Furthermore, although the scaffold constraint in VeGA-SCX is implemented as a soft conditioning signal rather than a rigid structural copy mechanism, it inherently biases the generative process toward scaffold retention. As a result, extensive exploration may be comparatively less pronounced than in purely semantic conditioning (VeGA-RX), reflecting a deliberate trade-off between precision-driven exploitation and exploratory diversity.

In addition, the framework is not designed as a free-text-to-molecule model. Unlike language-conditioned generative approaches, VeGA employs chemically explicit token conditioning rather than natural language prompts. This enhances interpretability and constraint fidelity but limits direct applicability in unconstrained natural language design scenarios.

Finally, it should be emphasized that the primary strength of this framework lies in leveraging explicit SMARTS-RX conditioning to ensure that the generated chemical space is populated with appropriate functional groups, thereby enabling the construction of focused libraries composed of chemically plausible candidates that potentially fit desired binding sites. Within this context, while molecular docking and MD simulations provide structural and energetic support for binding hypotheses, experimental biochemical and cellular assays are ultimately required to confirm binding affinity and functional activity of the proposed candidates. Accordingly, the present framework should be interpreted as a tool for hypothesis generation and library prioritization rather than a substitute for experimental validation.

## 5 Conclusions

In this study, we introduced a controllable and interpretable generative framework based on the VeGA architecture, designed to address the critical balance between exploration and exploitation in de novo drug design. The proposed models are designed to operate at different levels of design granularity, reflecting the evolving requirements of medicinal chemistry workflows.

Leveraging the recently established SMARTS-RX vocabulary, we developed two distinct models: VeGA-RX, conditioned on semantic functional descriptors, and VeGA-SCX, which integrates these semantic constraints with topological guidance via Bemis-Murcko scaffolds.

Our analysis demonstrates that, even in the unconditional setting, both models deliver performance comparable to state-of-the-art baselines. We identified a low-temperature sampling regime (*T* = 0.6) that acts as an intrinsic chemical filter, minimizing the inclusion of alert-prone functional groups and optimizing drug-likeness properties without requiring additional RL optimization.

In the context of targeted fine-tuning, the results confirm distinct operational roles. By leveraging the dual conditioning of scaffold and SMARTS-RX, VeGA-SCX effectively explores a chemical space proximal to the training distribution, prioritizing the recovery of known bioactive chemotypes. Conversely, VeGA-RX maintains the ability to explore a relevant bioactive space but with greater creativity, proving particularly effective in navigating disjointed chemical landscapes where rigid structural constraints might otherwise limit generative validity.

Furthermore, benchmarking against LDMol, a state-of-the-art diffusion model, highlights the suitability of our autoregressive framework under the evaluated conditioning setup.

Diffusion models often rely on ambiguous natural language prompts and may suffer from lower chemical validity in complex scenarios. In contrast, VeGA ensures strict adherence to precise structural inputs via the SMARTS-RX vocabulary and delivers superior medicinal chemistry quality with significantly lower computational latency.

The application to data-scarce targets, such as DCAF1 and WRN helicase, illustrated the potential for controlled *de novo* library generation in these challenging contexts. Nevertheless, important limitations must be acknowledged: unlike protein-conditioned generative models that learn to map protein sequences directly to ligand structures, VeGA-RX and VeGA-SCX do not inherently encode target information. They rely on the user, or a reference ligand, to define the appropriate chemical constraints (SMARTS/Scaffold). Therefore, they are not “black-box” target solvers but rather expert-guided engines for chemical space exploration. Their true value lies in coupling this controllable generation with downstream structure-based validation, providing a computational workflow to propose novel analogs with appropriate functional groups. These models are therefore intended as tools for hypothesis generation and prioritization within a broader medicinal chemistry workflow, rather than as standalone predictors of experimentally validated compounds. These molecules serve as chemically plausible candidates for in vitro and in vivo experimental validation, providing high-quality starting points for further optimization prior to synthesis.

To support reproducibility and facilitate community adoption, the complete workflow, including source code, pretrained models, and tutorials, is made freely available on GitHub. Overall, these results position VeGA-RX and VeGA-SCX as complementary tools that expand the controllability and applicability of generative models in medicinal chemistry (https://github.com/piedelre93/VeGA-RX).

## Supplementary Material



## Data Availability

The ChEMBL database (https://www.ebi.ac.uk/chembl/) is a public domain data resource. RDKit is available at https://zenodo.org/records/15605628. TensorFlow is available at https://www.tensorflow.org/. Schrödinger Suite (https://www.schrodinger.com), a licensed software suite for biomolecular simulation and analysis, was used for docking studies. PyMOL (https://pymol.org/), a molecular visualization tool distributed under a license, was used for displaying and analyzing 3D structures and for figure preparation. The SMARTS-RX code is freely available under the Apache license 2.0 at GitHub: https://github.com/MolecularAI/smartsrx/The code, data sets and fine-tuned models are freely available under the MITlicense at GitHub: https://github.com/piedelre93/VeGA-for-de-novo-design.

## Notes

Section S1. Mathematical formulation of the VeGA architecture and training protocol. Figure S1. The histograms report the frequency (%) of SMARTS-RX descriptors identified in A. The ChEMBL training data set, serving as the reference chemical space; B. Molecules generated by VeGA-RX at a sampling temperature of *T* = 1.0; and C. Molecules generated at *T* = 0.6. The comparison highlights the shift in functional group distribution induced by temperature scaling. Figure S2. Comparison of physicochemical property distributions between molecules generated by VeGA, VeGA-RX, VeGA-SCX, and the ChEMBL reference data set. Distributions include MW, log *P*, ring count, HBD/A, and rotatable bonds. Line styles/colors correspond to the following models: VeGA (dashed black), VeGA-RX (solid blue), VeGA-SCX (solid orange), and the ChEMBL reference data set (solid gray). The results demonstrate close alignment of the conditional models with the training distribution, with VeGA-SCX exhibiting stronger structural adherence and VeGA-RX exploring slightly expanded chemical space. Figure S3. Two-dimensional UMAP projections of the chemical space explored by the VeGA models across five pharmacological targets: (A) MAPK1, (B) GBA, (C) FXR, (D) PKM2, and (E) mTORC1. Training set molecules are shown as black points, VeGA-SCX-generated molecules as red points, and VeGA-RX-generated molecules as blue points. All data sets are projected into the same embedding space to enable direct comparison of chemical space coverage. VeGA-SCX distributions appear more tightly clustered around the training set manifold, whereas VeGA-RX exhibits broader dispersion toward adjacent chemical regions. Figure S4. Representative molecules generated under conditional design inspired by CHEMBL1356585. VeGA-SCX preserves the benzoxazole scaffold while introducing peripheral variations, whereas VeGA-RX generates structurally diverse multiring analogs consistent with the specified SMARTS constraints. The numerical identifiers shown beneath each 2D structure correspond to CAS Finder registry entries, confirming that the generated compounds exist in chemical databases and have been previously synthesized. Figure S5. Ligand heavy-atom root-mean-square deviation (RMSD) trajectories during 100 ns molecular dynamics (MD) simulations. Panels (A–C) show the structural stability of the top-ranked generated candidates (Top-1, Top-2, and Top-3) in complex with DCAF1 (PDB: 8F8E). Panels (D–F) display the top-ranked candidates (Top-4, Top-5, and Top-6) in complex with the WRN helicase (PDB: 8PFO). For each candidate, the four independent 100 ns replicates are represented by solid colored lines. The dashed lines denote the trajectories of the respective cocrystallized reference ligands: OICR-8268 for DCAF1 (A–C) and HRO761 for the WRN helicase (D–F). All RMSD values (Å) were calculated relative to the initial docked conformations. Table S1. Architectural and computational characteristics of the VeGA baseline and conditional variants. Reported metrics include number of Transformer layers, model dimension (d_model), feed-forward dimension, vocabulary size, total number of parameters, training time per epoch, total training time, and average generation time per molecule. The conditional VeGA-RX and VeGA-SCX models exhibit a substantial increase in parameter count relative to the baseline architecture. Table S2. Quantitative characterization of the curated ChEMBL pretraining corpus before and after application of the 140-token sequence-length filter, together with the corresponding properties of molecules generated by VeGA-RX and VeGA-SCX. The Precurated Set corresponds to the curated data set prior to length filtering, whereas the ChEMBL Set corresponds to the final filtered training set used for pretraining. Reported metrics include number of molecules, molecular weight, LogP, QED score, SA score, number of H-bond donors and acceptors, ring count, number of rotatable bonds, and number of unique scaffolds. Values are reported as mean ± standard deviation. Table S3. KS statistics and KL divergence values comparing the physicochemical property distributions of generated molecules (VeGA-RX and VeGA-SCX) against the ChEMBL reference data set. Reported properties include Molecular Weight, logP, QED, SA score, H-Bond donors and acceptors, rotatable bonds, and ring count. All comparisons yielded statistically significant differences (*p* < 0.001). Note: Due to the large sample sizes, *p*-values are uniformly <0.001; thus, the KS statistic serves as the primary indicator of distributional overlap (lower values indicate higher similarity). Table S4. Quantitative decomposition of QED components across temperature scaling regimes (*T* = 1.0 vs *T* = 0.6). Reported values include mean descriptor values, absolute variation (Δ), and statistical significance determined via one-way ANOVA (*p*-value). The analysis encompasses all eight constituent parameters: molecular weight, octanol–water partition coefficient (ALOGP), H-bond donors and acceptors, topological polar surface area (PSA), rotatable bonds, aromatic ring count, and structural alerts. Table S5. Quantitative comparison of SMARTS-RX category frequencies generated at *T* = 1.0 and *T* = 0.6. Reported values include absolute frequency (%), relative variation (Δ), and associated chemical implications. Lower temperature preferentially suppresses structural-alert-associated functional groups and highly polar moieties. Table S6. Frequency (%) of selected SMARTS-RX categories generated at sampling temperatures *T* = 0.6 and *T* = 1.0 across three independent runs (original + two additional replicates; *n* = 10,000 molecules per run). Values are reported as mean ± standard deviation. Categories are reported in the same order as Table S5 for direct comparability. Table S7. Summary of key protein–ligand interactions and mean occupancy (±SD) recorded over four independent 100 ns replicates. Occupancy represents the percentage of simulation time during which the interaction was maintained. Residues highlighted in red indicate interactions shared with the cocrystallized reference ligand, confirming the preservation of the experimental binding motif across the generated candidates (PDF)
