# Supplementary material for: VeGA-RX and VeGA-SCX: Controllable SMARTS-Guided Generative Transformers for Precision-Driven De Novo Drug Design
Source: J Chem Inf Model. 2026 Apr 17;66(9):5189–205. doi: 10.1021/acs.jcim.6c00535 (PMC13169354; doi:10.1021/acs.jcim.6c00535)
Supplement: Supplementary file 1 [file ci6c00535_si_001.pdf]

Supporting information for:

VeGA-RX and VeGA-SCX: Controllable SMARTS-Guided Generative Transformers for Precision-Driven de Novo Drug Design

Pietro Delre[a], Giada Bellofatto[a], Antonio Lavecchia[a]*

[a]*Department of Pharmacy, "Drug Discovery Laboratory", University of Naples Federico II, via Domenico Montesano 49, I-80131 Naples, Italy*

*Email: antonio.lavecchia@unina.it

Section S1. Mathematical Formulation of the VeGA Architecture and Training Protocol.

Figure S1. The histograms report the frequency (%) of SMARTS-RX descriptors identified in: A. The ChEMBL training dataset, serving as the reference chemical space; B. Molecules generated by VeGA-RX at a sampling temperature of T = 1.0; and C. Molecules generated at T = 0.6. The comparison highlights the shift in functional group distribution induced by temperature scaling.

Figure S2. Comparison of physicochemical property distributions between molecules generated by VeGA, VeGA-RX, VeGA-SCX, and the ChEMBL reference dataset. Distributions include MW, logP, ring count, HBD/A, and rotatable bonds. Line styles/colors correspond to the following models: VeGA (dashed black), VeGA-RX (solid blue), VeGA-SCX (solid orange), and the ChEMBL reference dataset (solid grey). The results demonstrate close alignment of the conditional models with the training distribution, with VeGA-SCX exhibiting stronger structural adherence and VeGA-RX exploring slightly expanded chemical space.

Figure S3. Two-dimensional UMAP projections of the chemical space explored by the VeGA models across five pharmacological targets: (A) MAPK1, (B) GBA, (C) FXR, (D) PKM2, and (E) mTORC1. Training set molecules are shown as black points, VeGA-SCX–generated molecules as red points, and VeGA-RX–generated molecules as blue points. All datasets are projected into the same embedding space to enable direct comparison of chemical space coverage. VeGA-SCX distributions appear more tightly clustered around the training set manifold, whereas VeGA-RX exhibits broader dispersion toward adjacent chemical regions.

Figure S4. Representative molecules generated under conditional design inspired by CHEMBL1356585. VeGA-SCX preserves the benzoxazole scaffold while introducing peripheral variations, whereas VeGA-RX generates structurally diverse multi-ring analogs consistent with the specified SMARTS constraints. The numerical identifiers shown beneath each 2D structure correspond to CAS Finder registry entries, confirming that the generated compounds exist in chemical databases and have been previously synthesized.

Figure S5. Ligand heavy-atom root-mean-square deviation (RMSD) trajectories during 100 ns molecular dynamics (MD) simulations. Panels (A–C) show the structural stability of the top-ranked generated candidates (Top-1, Top-2, and Top-3) in complex with DCAF1 (PDB: 8F8E). Panels (D–F) display the top-ranked candidates (Top-4, Top-5, and Top-6) in complex with the WRN helicase (PDB: 8PFO). For each candidate, the four independent 100 ns replicates are represented by solid colored lines. The dashed lines denote the trajectories of the respective co-crystallized reference ligands: OICR-8268 for DCAF1 (A–C) and HRO761 for the WRN helicase (D–F). All RMSD values (Å) were calculated relative to the initial docked conformations.

Table S1. Architectural and computational characteristics of the VeGA baseline and conditional variants. Reported metrics include number of Transformer layers, model dimension (d_model), feed-forward dimension, vocabulary size, total number of parameters, training time per epoch, total training time, and average generation time per molecule. The conditional VeGA-RX and VeGA-SCX models exhibit a substantial increase in parameter count relative to the baseline architecture.

Table S2. Quantitative characterization of the curated ChEMBL pretraining corpus before and after application of the 140-token sequence-length filter, together with the corresponding properties of molecules generated by VeGA-RX and VeGA-SCX. The Pre-curated Set corresponds to the curated

dataset prior to length filtering, whereas the ChEMBL Set corresponds to the final filtered training set used for pretraining. Reported metrics include number of molecules, molecular weight, LogP, QED score, SA score, number of H-bond donors and acceptors, ring count, number of rotatable bonds, and number of unique scaffolds. Values are reported as mean ± standard deviation.

Table S3. KS statistics and KL divergence values comparing the physicochemical property distributions of generated molecules (VeGA-RX and VeGA-SCX) against the ChEMBL reference dataset. Reported properties include Molecular Weight, logP, QED, SA score, H-Bond donors and acceptors, rotatable bonds, and ring count. All comparisons yielded statistically significant differences ($p < 0.001$). Note: Due to the large sample sizes, p-values are uniformly <0.001; thus, the KS statistic serves as the primary indicator of distributional overlap (lower values indicate higher similarity).

Table S4. Quantitative decomposition of QED components across temperature scaling regimes (T=1.0 vs T=0.6). Reported values include mean descriptor values, absolute variation (Δ), and statistical significance determined via one-way ANOVA (p-value). The analysis encompasses all eight constituent parameters: Molecular Weight, octanol-water partition coefficient (ALOGP), H-Bond donors and acceptors, topological polar surface area (PSA), rotatable bonds, aromatic ring count, and structural alerts.

Table S5. Quantitative comparison of SMARTS-RX category frequencies generated at T = 1.0 and T = 0.6. Reported values include absolute frequency (%), relative variation (Δ), and associated chemical implications. Lower temperature preferentially suppresses structural-alert–associated functional groups and highly polar moieties.

Table S6. Frequency (%) of selected SMARTS-RX categories generated at sampling temperatures T = 0.6 and T = 1.0 across three independent runs (original + two additional replicates; n = 10,000 molecules per run). Values are reported as mean ± standard deviation. Categories are reported in the same order as Table S5 for direct comparability.

Table S7. Summary of key protein-ligand interactions and mean occupancy (± SD) recorded over four independent 100 ns replicates. Occupancy represents the percentage of simulation time during which the interaction was maintained. Residues highlighted in red indicate interactions shared with the co-crystallized reference ligand, confirming the preservation of the experimental binding motif across the generated candidates.

Section S1. Mathematical Formulation of the VeGA Architecture and Training Protocol.

This section provides comprehensive mathematical details of the Transformer architecture and the optimization schedule employed in the VeGA models, complementing the description provided in Section 2.3 of the main manuscript.

The model relies on scaled dot-product attention. To ensure the autoregressive generation property (preventing the model from attending to future tokens), a causal mask matrix M is applied. The attention function maps a Query (Q), Key (K), and Value (V) matrix to an output, computed as follows:

$$Attention(Q,K,V) = softmax\left(\frac{QK^T}{\sqrt{d_k}} + M\right)V \quad \textbf{(4)}$$

where Attention (Q, K, V) is the attention function that produces a contextual representation by combining Query (Q), Key (K), and Value (V) matrices. A scaling factor ($\sqrt{d_k}$), stabilizes gradients during training, while a Masking matrix (M) ensures causality.

Positional information was encoded using fixed sinusoidal positional encodings:

$$PE_{(pos,2i)} = sin\left(\frac{pos}{10000^{\frac{2i}{d_{model}}}}\right), PE_{(pos,2i+1)} = cos\left(\frac{pos}{10000^{\frac{2i}{d_{model}}}}\right) \quad \textbf{(5)}$$

where $PE_{(pos,2i(+1))}$ is the positional encoding function that assigns unique vectors to token positions. pos: Position index of the token in the sequence. i: Dimension index in the embedding vector. d_{model}: Dimensionality of the model's embeddings. sin/cos functions: Mathematical operations creating unique position patterns with consistent relative distances between tokens.

Training protocol. We trained using Adam with the Transformer "inverse square-root / warmup" schedule:[40]

$$\boldsymbol{lr(t) = d_{model}^{-0.5} * \min(t^{-0.5}, t * warmup\_steps^{-1.5})} \quad (6)$$

where $lr(t)$ is the learning rate at step t; d_{model} represents the embedding dimensionality (512 in VeGA); and warmup_steps defines the initial warmup period. The $\min()$ function controls the transition from the linear warmup to the inverse-square-root decay phase. Data were split into 90% training and 10% validation sets. The objective was sparse categorical cross-entropy with an explicit mask excluding <PAD> tokens from gradient updates. Training ran for up to 100 epochs.

A

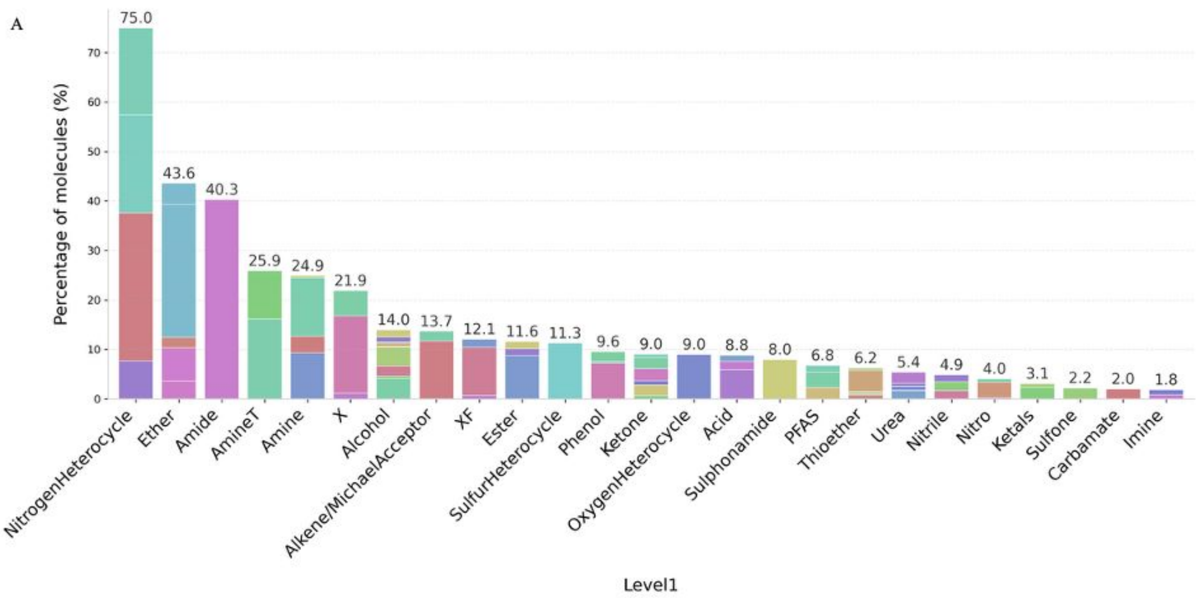
Percentage of molecules (%)
Level1
75.0
43.6
40.3
25.9
24.9
21.9
14.0
13.7
12.1
11.6
11.3
9.6
9.0
9.0
8.8
8.0
6.8
6.2
5.4
4.9
4.0
3.1
2.2
2.0
1.8
NitrogenHeterocycle
Ether
Amide
AmineT
Amine
X
Alcohol
Alkene/MichaelAcceptor
XF
Ester
SulfurHeterocycle
Phenol
Ketone
OxygenHeterocycle
Acid
Sulphonamide
PFAS
Thioether
Urea
Nitrile
Nitro
Ketals
Sulfone
Carbamate
Imine

B

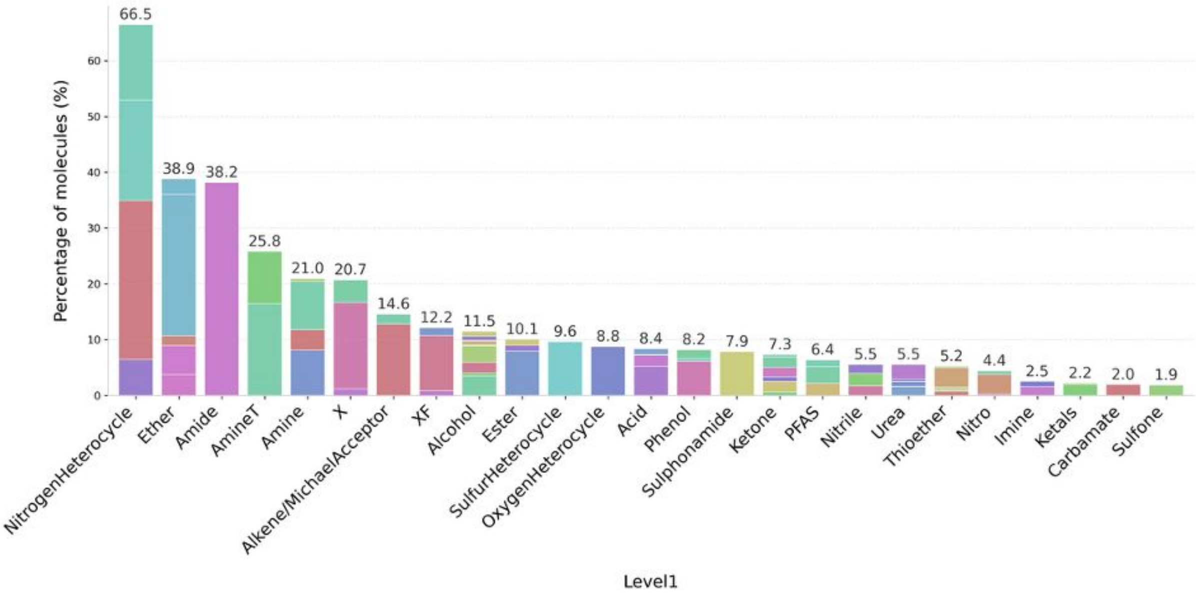
Percentage of molecules (%)
Level1
66.5
38.9
38.2
25.8
21.0
20.7
14.6
12.2
11.5
10.1
9.6
8.8
8.4
8.2
7.9
7.3
6.4
5.5
5.5
5.2
4.4
2.5
2.2
2.0
1.9
NitrogenHeterocycle
Ether
Amide
AmineT
Amine
X
Alkene/MichaelAcceptor
XF
Alcohol
Ester
SulfurHeterocycle
OxygenHeterocycle
Acid
Phenol
Sulphonamide
Ketone
PFAS
Nitrile
Urea
Thioether
Nitro
Imine
Ketals
Carbamate
Sulfone

C

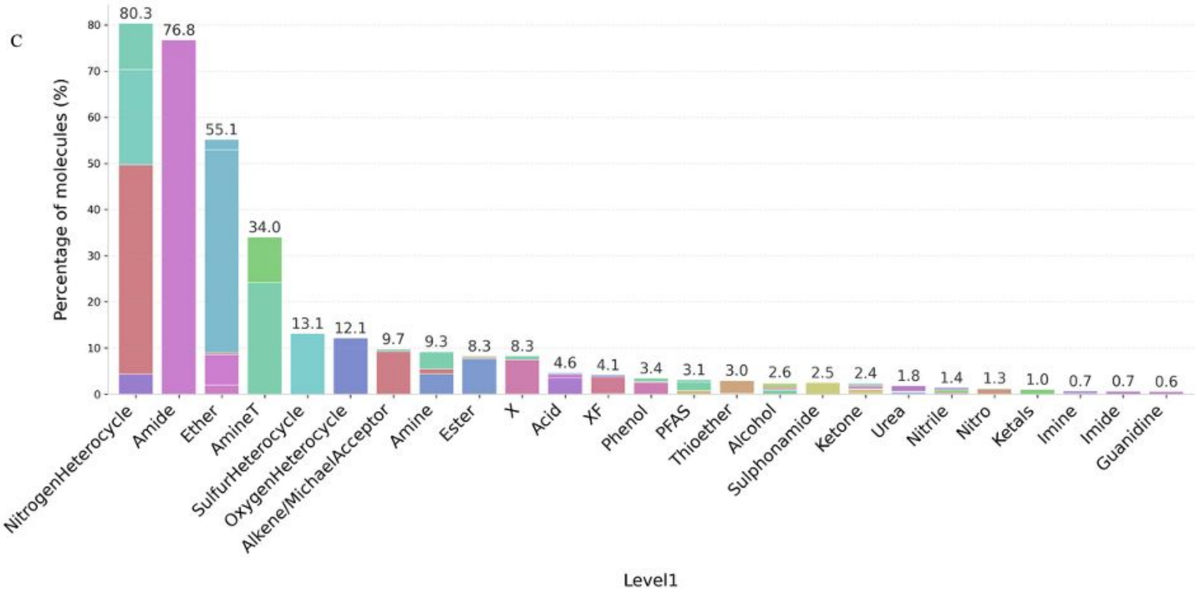
Percentage of molecules (%)
Level1
80.3
76.8
55.1
34.0
13.1
12.1
9.7
9.3
8.3
8.3
4.6
4.1
3.4
3.1
3.0
2.6
2.5
2.4
1.8
1.4
1.3
1.0
0.7
0.7
0.6
NitrogenHeterocycle
Amide
Ether
AmineT
SulfurHeterocycle
OxygenHeterocycle
Alkene/MichaelAcceptor
Amine
Ester
X
Acid
XF
Phenol
PFAS
Thioether
Alcohol
Sulphonamide
Ketone
Urea
Nitrile
Nitro
Ketals
Imine
Imide
Guanidine

Figure S1. The histograms report the frequency (%) of SMARTS-RX descriptors identified in: (A) the ChEMBL training dataset, serving as the reference chemical space; (B) molecules generated by VeGA-RX at a sampling temperature of T = 1.0; and (C) molecules generated at T = 0.6. The comparison highlights the shift in functional group distribution induced by temperature scaling.

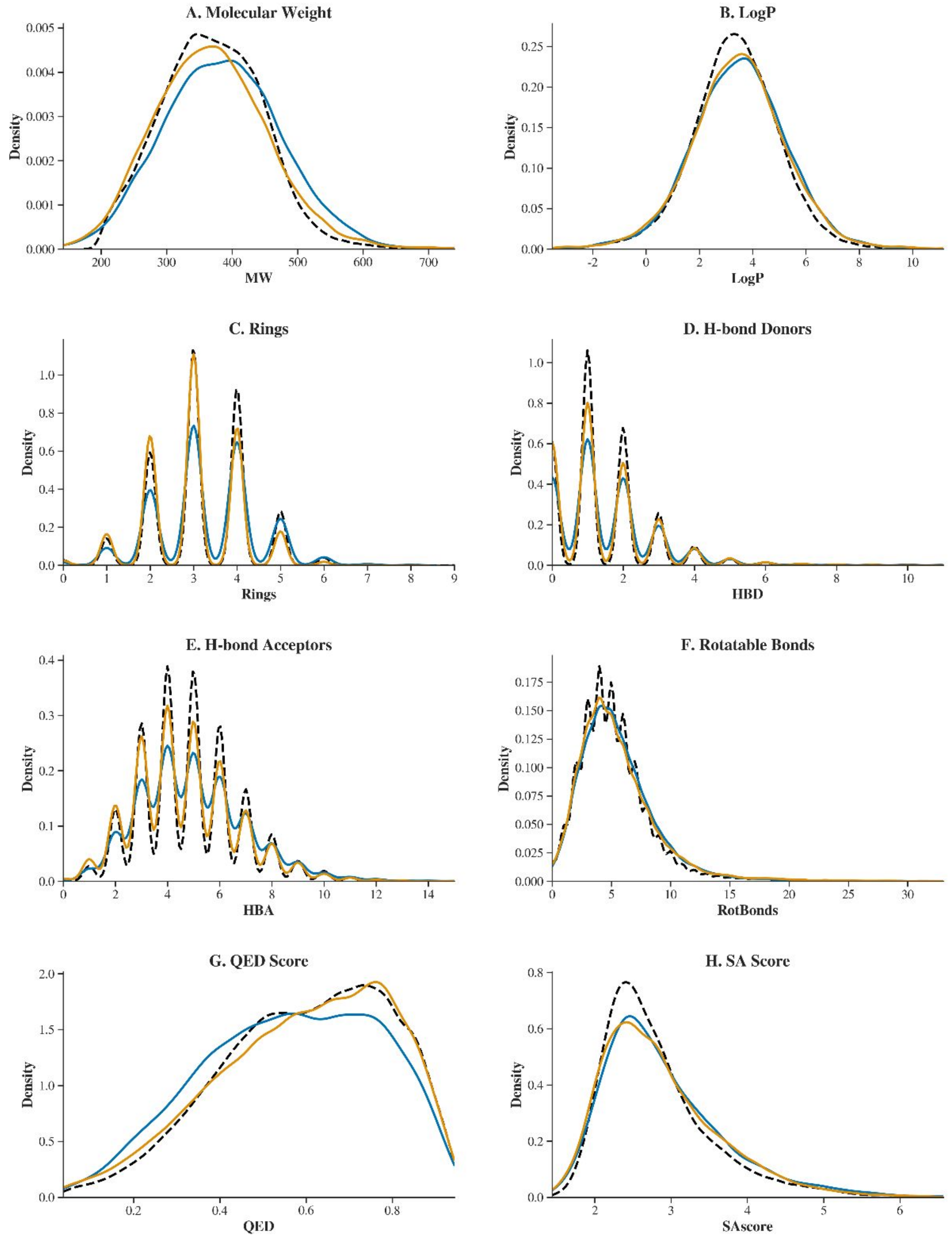

Figure S2. Comparison of physicochemical property distributions between molecules generated by VeGA, VeGA-RX, VeGA-SCX, and the ChEMBL reference dataset. Distributions include MW, logP, ring count, HBD/A, and rotatable bonds. Line styles/colors correspond to the following models: VeGA (dashed black), VeGA-RX (solid blue), VeGA-SCX (solid orange), and the ChEMBL reference dataset (solid grey). The results demonstrate close alignment of the conditional models with the training distribution, with VeGA-SCX exhibiting stronger structural adherence and VeGA-RX exploring slightly expanded chemical space.

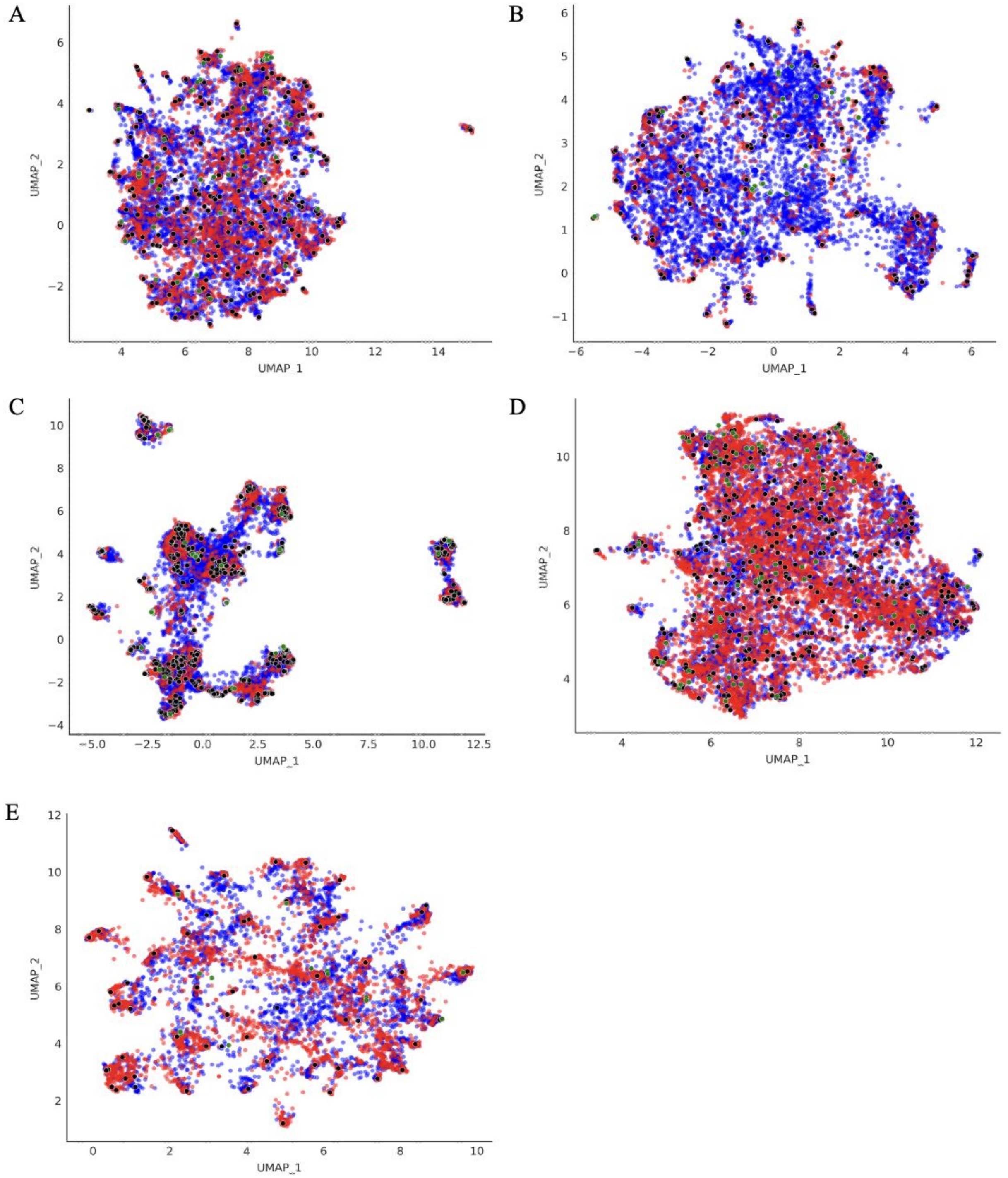

Figure S3. Two-dimensional UMAP projections of the chemical space explored by the VeGA models across five pharmacological targets: (A) MAPK1, (B) GBA, (C) FXR, (D) PKM2, and (E) mTORC1. Training set molecules are shown as black points, holdout set molecules as green points, VeGA-SCX–generated molecules as red points, and VeGA-RX–generated molecules as blue points. All datasets are projected into the same embedding space to enable direct comparison of chemical space

coverage. VeGA-SCX distributions appear more tightly clustered around the training set manifold, whereas VeGA-RX exhibits broader dispersion toward adjacent chemical regions.

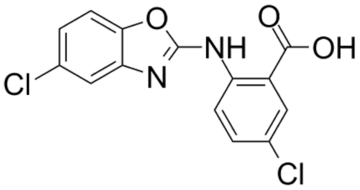

CHEMBL1356585
Scaffold : C1=CC2=C(C=C1)OC=N2
SMARTS-RX: [Acid_Aromatic, Amine_AcyclicSecondary_Aromatic-Heteroaromatic, Heterocycle5_O, Heterocycle5_polyHet, X-Chloride_Phe, X-Chloride_PheHet5]

VeGA-RX

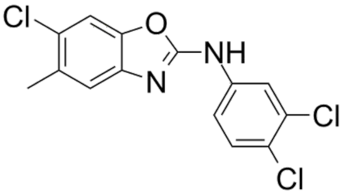

2334318-19-1

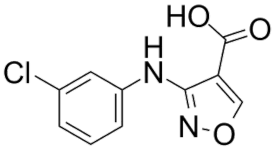

2111164-61-3

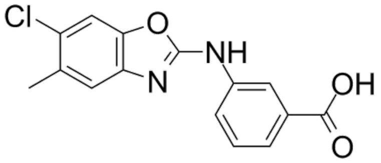

2343921-03-7

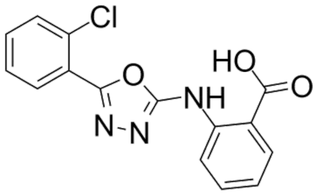

1368365-31-4

VeGA-SCX

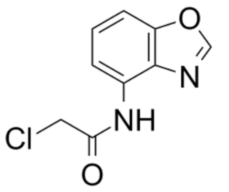

1638835-05-8

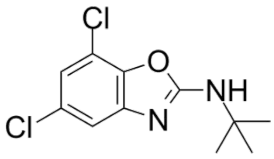

1427468-73-2

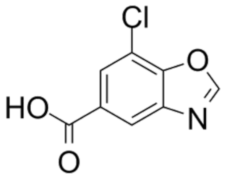

1378841-78-1

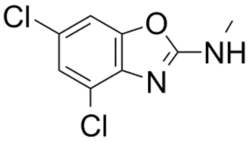

1092044-51-3

Figure S4. Representative molecules generated under conditional design inspired by CHEMBL1356585. VeGA-SCX preserves the benzoxazole scaffold while introducing peripheral variations, whereas VeGA-RX generates structurally diverse multi-ring analogs consistent with the specified SMARTS constraints. The numerical identifiers shown beneath each 2D structure correspond to CAS Finder registry entries, confirming that the generated compounds exist in chemical databases and have been previously synthesized.

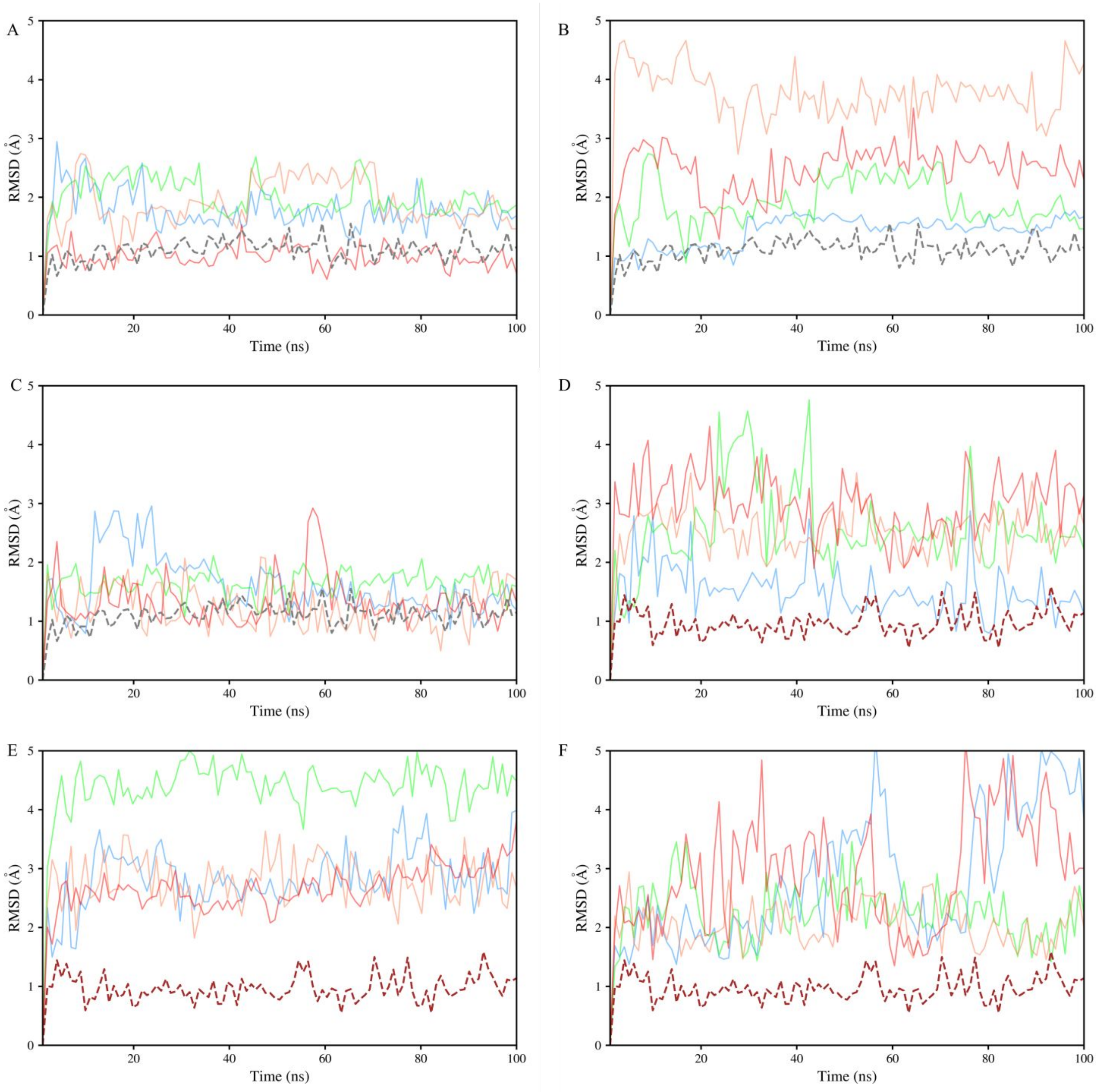

Figure S5. Ligand heavy-atom root-mean-square deviation (RMSD) trajectories during 100 ns molecular dynamics (MD) simulations. Panels (A–C) show the structural stability of the top-ranked generated candidates (Top-1, Top-2, and Top-3) in complex with DCAF1 (PDB: 8F8E). Panels (D–F) display the top-ranked candidates (Top-4, Top-5, and Top-6) in complex with the WRN helicase (PDB: 8PFO). For each candidate, the four independent 100 ns replicates are represented by solid colored lines. The dashed lines denote the trajectories of the respective co-crystallized reference ligands : OICR-8268 for DCAF1 (A–C) and HRO761 for the WRN helicase (D–F). All RMSD values (Å) were calculated relative to the initial docked conformations.

Table S1. Architectural and computational characteristics of the VeGA baseline and conditional variants. Reported metrics include number of Transformer layers, model dimension (d_model), feed-forward dimension, vocabulary size, total number of parameters, training time per epoch, total training time, and average generation time per molecule. The conditional VeGA-RX and VeGA-SCX models exhibit a substantial increase in parameter count relative to the baseline architecture.

Model	# Layers	d_model	FFN Dim	Vocabulary	Total Params	Time per Epoch	Total Training Time	Generation Time	Parameters	Hardware
VeGA (Baseline)	4	100	300	36	~0.8M	~7.5 min	~7 h (50 epochs)	~20 ms/mol	Adam (β_1=0.9, β_2=0.999, $\varepsilon=1\times10^{-7}$); warmup=4,000 steps; batch size=128 (pretraining)	Single NVIDIA RTX A2000 (12 GB VRAM)
VeGA-RX	6	512	2048	370	50,796,914 (~50.8M)	~11.5 min	~38 h (200 epochs)	~220 ms/mol	Adam (β_1=0.9, β_2=0.999, $\varepsilon=1\times10^{-7}$); warmup=4,000 steps; batch size=32 (pretraining)	Single NVIDIA RTX A2000 (12 GB VRAM)
**VeGA-SCX**	6	512	2048	370	50,796,914 (~50.8M)	~11.5 min	~38 h (200 epochs)	~310 ms/mol	Adam (β_1=0.9, β_2=0.999, $\varepsilon=1\times10^{-7}$); warmup=4,000 steps; batch size=32 (pretraining)	Single NVIDIA RTX A2000 (12 GB VRAM)

Table S2. Quantitative characterization of the curated ChEMBL pretraining corpus before and after application of the 140-token sequence-length filter, together with the corresponding properties of molecules generated by VeGA-RX and VeGA-SCX. The Pre-curated Set corresponds to the curated dataset prior to length filtering, whereas the ChEMBL Set corresponds to the final filtered training set used for pretraining. Reported metrics include number of molecules, molecular weight, LogP, QED score, SA score, number of H-bond donors and acceptors, ring count, number of rotatable bonds, and number of unique scaffolds. Values are reported as mean ± standard deviation.

**Property**	**Pre-curated Set**	**ChEMBL Set**	**VeGA-SCX**	**VeGA-RX**
Number of molecules	1,092,285	891,004	30,000	30,000
Molecular Weight	405.41 ± 107.24	370.77 ± 77.75	368.90 ± 89.48	385.03 ± 90.32
LogP	3.59 ± 1.82	3.36 ± 1.67	3.48 ± 1.87	3.49 ± 1.79
QED Score	0.55 ± 0.22	0.60 ± 0.19	0.60 ± 0.20	0.56 ± 0.20
SA Score	2.93 ± 0.82	2.81 ± 0.73	2.86 ± 0.78	2.91 ± 0.81
H-bond Donors	1.56 ± 1.41	1.43 ± 1.26	1.40 ± 1.33	1.50 ± 1.36
H-bond Acceptors	5.25 ± 2.21	4.84 ± 1.92	4.69 ± 1.99	4.99 ± 2.06
Ring Count	3.56 ± 1.33	3.22 ± 1.09	3.02 ± 1.05	3.31 ± 1.14
Rotatable Bonds	5.74 ± 3.44	5.24 ± 3.18	5.55 ± 3.73	5.53 ± 3.31
Number of Unique Scaffolds	364,673	286,136	13,631	15,531

Table S3. KS statistics and KL divergence values comparing the physicochemical property distributions of generated molecules (VeGA-RX and VeGA-SCX) against the ChEMBL reference dataset. Reported properties include Molecular Weight, logP, QED, SA score, H-Bond donors and acceptors, rotatable bonds, and ring count. All comparisons yielded statistically significant differences ($p < 0.001$). Note: Due to the large sample sizes, p-values are uniformly <0.001; thus, the KS statistic serves as the primary indicator of distributional overlap (lower values indicate higher similarity).

Model	Property	Ks Statistic	Kl Divergence	P-Value
**VEGA-RX**	Molecular Weight	0.0866	0.0509	< 0.001
	LogP	0.0453	0.0167	< 0.001
	QED	0.0753	0.0196	< 0.001
	SA Score	0.0615	0.0182	< 0.001
	H-Bond Donors	0.0313	0.0104	< 0.001
	H-Bond Acceptors	0.0334	0.0104	< 0.001
	Rotatable Bonds	0.0456	0.0141	< 0.001
	Ring Count	0.0329	0.0064	< 0.001
**VEGA-SCX**	Molecular Weight	0.0353	0.0240	< 0.001
	LogP	0.0376	0.0119	< 0.001
	QED	0.0199	0.0044	< 0.001
	SA Score	0.0464	0.0178	< 0.001
	H-Bond Donors	0.0415	0.0079	< 0.001
	H-Bond Acceptors	0.0436	0.0079	< 0.001
	Rotatable Bonds	0.0397	0.0074	< 0.001
	Ring Count	0.0825	0.0233	< 0.001

Table S4. Quantitative decomposition of QED components across temperature scaling regimes (T=1.0 vs T=0.6). Reported values include mean descriptor values, absolute variation (Δ), and statistical significance determined via one-way ANOVA (p-value). The analysis encompasses all eight constituent parameters: Molecular Weight, octanol-water partition coefficient (ALOGP), H-Bond donors and acceptors, topological polar surface area (PSA), rotatable bonds, aromatic ring count, and structural alerts.

Property	T = 1.0	T = 0.6	Variation (Δ)	p-value (ANOVA)
QED	0.565	0.652	+0.087	$1.51e^{-199}$
Molecular Weight	385.03	365.05	-19.98	$2.36e^{-58}$
ALOGP	3.49	3.56	+0.07	$6.72e^{-03}$
H-Bond acceptors	4.64	4.24	-0.40	$4.40e^{-51}$
H-Bond donors	1.50	1.25	-0.25	$3.44e^{-41}$
PSA (Å²)	76.52	68.17	-8.35	$1.24e^{-67}$
Rotatable bonds	5.53	5.79	+0.26	$6.80e^{-06}$
Aromatic ring	2.42	2.57	+0.15	$2.46e^{-22}$
Structural alerts	0.935	0.457	-0.478	$3.72e^{-234}$

Table S5. Quantitative comparison of SMARTS-RX category frequencies generated at T = 1.0 and T = 0.6. Reported values include absolute frequency (%), relative variation (Δ), and associated chemical implications. Lower temperature preferentially suppresses structural-alert–associated functional groups and highly polar moieties.

SMARTS Category	Frequency at T=1.0 (%)	Frequency at T=0.6 (%)	Variation (Δ)	Chemical Implication
Halogens (Category X)	29.67	12.11	- 17.56	Reduction of unspecific halogenation (MW/LogP control)
Amines	19.42	8.96	- 10.46	Reduction of basic centers and polarity
Alcohols	9.37	2.13	- 7.25	Reduction of polar H-bond donors
Sulphonamides	7.86	2.49	- 5.37	Removal of heavy, polar moieties
Michael Acceptors	14.20	9.64	- 4.57	Suppression of reactive/toxic alerts
Nitro Groups	4.33	1.27	- 3.06	Suppression of mutagenic/toxic alerts
Imines	2.48	0.66	- 1.82	Suppression of hydrolytically unstable groups

Table S6. Frequency (%) of selected SMARTS-RX categories generated at sampling temperatures T = 0.6 and T = 1.0 across three independent runs (original + two additional replicates; n = 10,000 molecules per run). Values are reported as mean ± standard deviation. Categories are reported in the same order as Table S5 for direct comparability.

SMARTS Category	Frequency at T = 1.0 (%)	Frequency at T = 0.6 (%)	Variation (Δ)	Chemical Implication
Halogens (Category X)	30.32 ± 0.07	12.68 ± 0.09	−17.64 ± 0.02	Reduction of unspecific halogenation (MW/LogP control)
Amines	20.95 ± 0.50	10.39 ± 0.06	−10.56 ± 0.43	Reduction of basic centers and polarity
Alcohols	9.18 ± 0.16	2.28 ± 0.01	−6.90 ± 0.15	Reduction of polar H-bond donors
Sulphonamides	7.99 ± 0.12	3.03 ± 0.25	−4.96 ± 0.13	Removal of heavy, polar moieties
Michael Acceptors	13.34 ± 0.08	9.43 ± 0.09	−3.91 ± 0.01	Suppression of reactive/toxic alerts
Nitro Groups	3.64 ± 0.17	1.02 ± 0.01	−2.62 ± 0.17	Suppression of mutagenic/toxic alerts
Imines	2.32 ± 0.10	0.66 ± 0.08	−1.66 ± 0.15	Suppression of hydrolytically unstable groups

Table S7. Summary of key protein-ligand interactions and mean occupancy (± SD) recorded over four independent 100 ns replicates. Occupancy represents the percentage of simulation time during which the interaction was maintained. Residues highlighted in red indicate interactions shared with the co-crystallized reference ligand, confirming the preservation of the experimental binding motif across the generated candidates.

TARGET	CANDIDATE	RESIDUE	INTERACTION TYPE	OCCUPANCY (%)
**DCAF1**	**Top-1**	Arg1298	Hydrogen bond	100.0 ± 0.0
		Phe1330	pi-p stacking	73.1 ± 21.0
		Phe1101	Halogen-hydrogen bond	69.0 ± 20.0
		Asp1356	Hydrogen bond	100 ± 0.0
	**Top-2**	Arg1225	Cation-pi	55.8 ± 4.3
		Asp1356	Hydrogen bond	88.5 ± 10.5
	**Top-3**	Arg1298	Hydrogen bond	99.0 ± 1.0
		Phe1330	pi-pi stacking	72.8 ± 4.3
		Asp1356	Hydrogen bond	99.0 ± 1.0
**WRN**	**Top-4**	Glu918	Hydrogen bond	70.8 ± 11.2
		Arg732	Hydrogen bond	100.0 ± 0.0
		Arg732	Cation-pi	56.5 ± 5.0
	**Top-5**	Tyr849	pi-pi stacking	55.75 ± 7.85
		Phe730	pi-pi stacking	67.00 ± 13.7
		Arg732	Hydrogen bond	91.50 ± 14.3
		Arg732	Cation-pi	85.50 ± 14.7
	**Top-6**	Phe730	Halogen bond (backbone)	67.00 ± 13.7
		Glu918	Hydrogen bond	99.3 ± 0.3
		Arg732	Hydrogen bond	100.0 ± 0.0
		Arg732	Cation-pi	78.50 ± 14.55
